# Computational Analysis of the Hypothalamic Control of Food Intake

**DOI:** 10.3389/fncom.2016.00027

**Published:** 2016-04-26

**Authors:** Shayan Tabe-Bordbar, Thomas J. Anastasio

**Affiliations:** Computational Neurobiology Laboratory, Department of Molecular and Integrative Physiology, Beckman Institute, University of Illinois at Urbana-ChampaignUrbana, IL, USA

**Keywords:** feeding, ingestive behavior, hypothalamus, neural network, computer model

## Abstract

Food-intake control is mediated by a heterogeneous network of different neural subtypes, distributed over various hypothalamic nuclei and other brain structures, in which each subtype can release more than one neurotransmitter or neurohormone. The complexity of the interactions of these subtypes poses a challenge to understanding their specific contributions to food-intake control, and apparent consistencies in the dataset can be contradicted by new findings. For example, the growing consensus that arcuate nucleus neurons expressing Agouti-related peptide (AgRP neurons) promote feeding, while those expressing pro-opiomelanocortin (POMC neurons) suppress feeding, is contradicted by findings that low AgRP neuron activity and high POMC neuron activity can be associated with high levels of food intake. Similarly, the growing consensus that GABAergic neurons in the lateral hypothalamus suppress feeding is contradicted by findings suggesting the opposite. Yet the complexity of the food-intake control network admits many different network behaviors. It is possible that anomalous associations between the responses of certain neural subtypes and feeding are actually consistent with known interactions, but their effect on feeding depends on the responses of the other neural subtypes in the network. We explored this possibility through computational analysis. We made a computer model of the interactions between the hypothalamic and other neural subtypes known to be involved in food-intake control, and optimized its parameters so that model behavior matched observed behavior over an extensive test battery. We then used specialized computational techniques to search the entire model state space, where each state represents a different configuration of the responses of the units (model neural subtypes) in the network. We found that the anomalous associations between the responses of certain hypothalamic neural subtypes and feeding are actually consistent with the known structure of the food-intake control network, and we could specify the ways in which the anomalous configurations differed from the expected ones. By analyzing the temporal relationships between different states we identified the conditions under which the anomalous associations can occur, and these stand as model predictions.

## Introduction

Eat when hungry, stop when satisfied. It would seem that nothing could be simpler than food-intake control. Indeed, early researchers studying the neural control of food intake believed that a single area, the lateral hypothalamus, was solely responsible for feeding (Anand and Brobeck, 1951; Grossman, 1960). Later work refined the role of the lateral hypothalamus and suggested that the ventromedial hypothalamus (VMH) could also be involved (Powley and Keesey, 1970). Recent work using sophisticated chemogenetic and optogenetic techniques reveals that food-intake control is mediated by multiple hypothalamic nuclei as well as striatal, midbrain, and hindbrain structures, and it involves complex interactions among many neural subtypes that vary in their responses to different hormones and also vary in the neurotransmitters and neurohormones they release onto each other (Keesey and Powley, 2008; Atasoy et al., 2012; Sohn et al., 2013; Sternson and Atasoy, 2014). These experiments present a complex dataset that is made all the more bewildering by new findings that contradict current understanding. For example, in the arcuate nucleus, neurons expressing Agouti-related peptide (AgRP neurons) generally promote feeding, while those expression pro-opiomelanocortin (POMC neurons) generally suppress feeding, but this tidy view is challenged by new findings that robust feeding can occur when AgRP neuron activity is decreased but that of POMC neurons is increased (Chen et al., 2015). In the lateral hypothalamus a similar conundrum involves GABAergic neurons that generally suppress feeding, but new findings suggest that they can promote feeding as well (Leinninger et al., 2009, 2011; Feifel et al., 2010; Laque et al., 2013; Opland et al., 2013; Goforth et al., 2014; Wu et al., 2015). These contradictions indicate that our current understanding of food-intake control is limited, but leave open the possibility that the anomalous findings concerning specific neural subtypes could be reconciled when they are considered in the context of the entire network. We explore this possibility computationally.

Our approach is to model the interactions among the hypothalamic, and some other, neural subtypes involved in food intake control and to use specialized computational tools to analyze those interactions. Only those interactions that have been well-documented in the literature as directly involved in food-intake control are included in the model (see section Neurobiological Basis of the Model). Nevertheless, a key aspect of our approach is the realization that neurons not included in the model can affect the responses of neurons that are included, and so can influence food-intake control. Our goal is to explore the subset of possible response configurations that are consistent with a limited number of plausible modulations of the interactions represented in the model. Model analysis exploits powerful computational methods based on declarative programming that facilitate enumeration of the entire model state space, which for the food-intake control model is the set of all allowed configurations (or network-wide patterns) of the responses of the neural subtypes represented in the model. We then apply tools known as state-space search and temporal-logic model-checking (Monin and Hinchey, 2003; Huth and Ryan, 2004), both to search for response configurations that satisfy certain criteria, and to determine temporal relationships between specific response patterns. This computational analysis is the first of its kind in the neuroscience of food-intake control.

Our analysis is focused on anomalous findings concerning specific hypothalamic neural subtypes that contradict the consensus view of the roles they play in food-intake control. By searching the space of response configurations we show that the anomalous findings are actually consistent with known interactions as represented in the model, and we identify specific response patterns that distinguish anomalous from expected configurations. These modeling results illustrate how contradictory findings on a few neural subtypes can be reconciled by viewing those subtypes as part of a larger network that can have many different response configurations. Then by analyzing the temporal relationships between various configurations we identify specific response patterns among other hypothalamic neural subtypes that could allow the anomalous associations to occur in the model. These predicted response patterns could be tested experimentally.

## Methods

**Figure 1:**
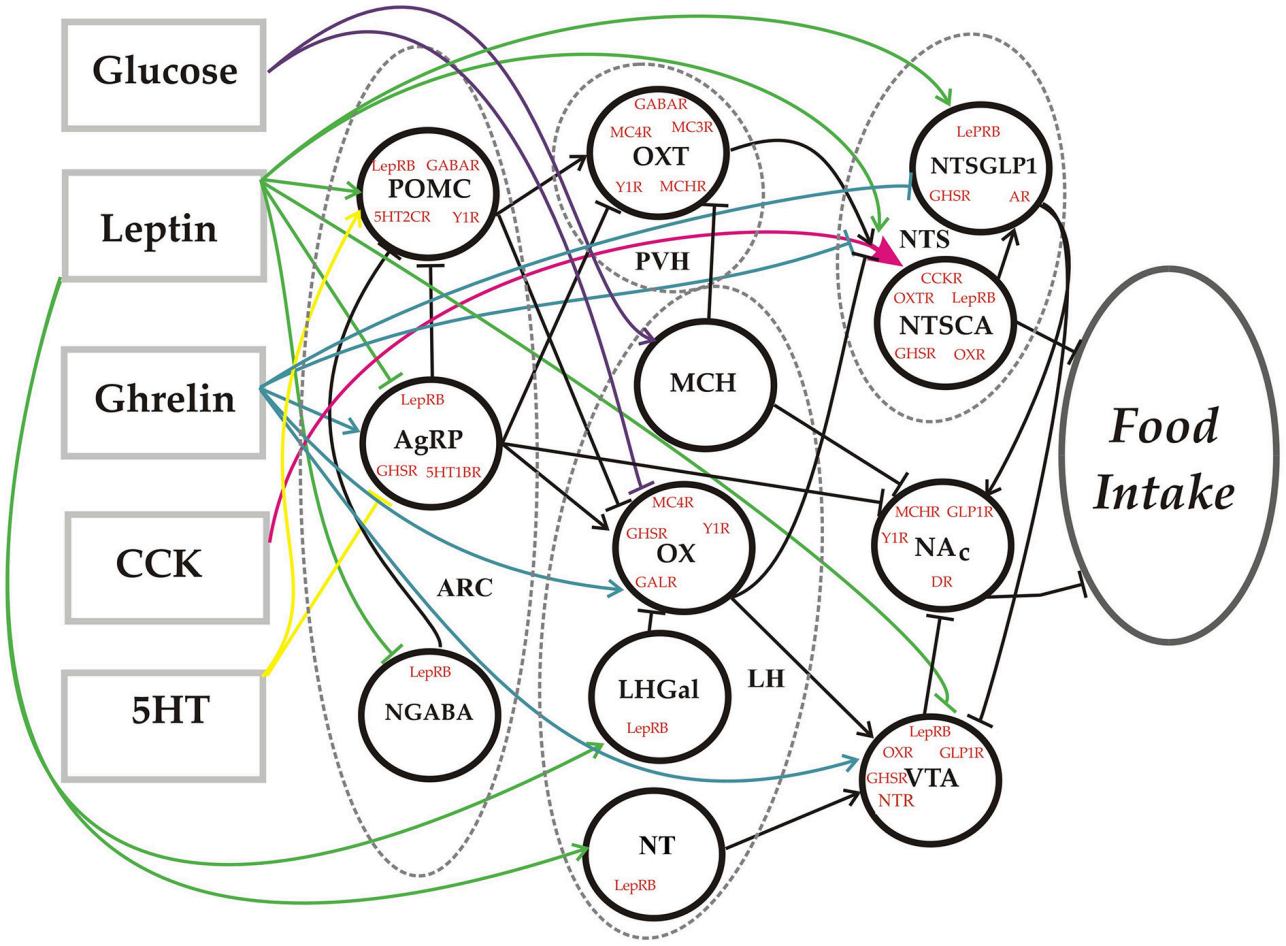
**Network diagram**. Schematic representation of the food-intake control network model. Dashed lines define the borders of specific hypothalamic or brainstem nuclei. Each circle denotes a unit in the network model that represents a distinct neural subtype defined in terms of its location, the neurotransmitters/neurohormones it releases, and the receptors it expresses. The rectangles represent substances that constitute the inputs to the model. The output is the level of food intake. Connections between model elements can be excitatory (arrow) or inhibitory (tee).

The model takes the form of a feedforward neural network (Figure 1). Each neural element (unit) represents all of the neurons of a given subtype as defined anatomically by their location (e.g., Arc) and neurochemically by the transmitters they release (e.g., AgRP, NPY, and GABA) (see Table 1 for all abbreviations). The network essentially transforms the levels of a set of feeding-related substances into an associated level of food intake. The response of each unit is determined by the strengths of its receptors for various neurotransmitters or neurohormones, and by its own intrinsic bias. The receptor strengths and unit biases (i.e., the parameters) are set using an optimization procedure so that the behavior of the model matches that of the real system over a range of inputs and observed outputs that we call the truth table (Table 2). The truth table specifies the behavior that the model is required to reproduce, and agreement between the model and the truth table signifies that the model is a valid representation of the food-intake control network. The entire space of response configurations of the validated model can then be searched for response patterns that satisfy specific criteria, and can also be analyzed to determine antecedent-consequent relationships between specific response patterns.

**Table 1:** **Table of abbreviations**.

**Full name**	**Standard abbreviation**	**Variable name**
5-hydroxytryptamine (serotonin)	5HT	FHT
5-hydroxytryptamine receptor type 1B	5HT1B receptor	FHT1BR
5-hydroxytryptamine receptor type 2C	5HT2C receptor	FHT2CR
Adrenergic receptors	n/a	AR
Agouti-related peptide	AgRP	AgRP
Alpha-melanocortin stimulating hormone	α-MSH	aMSH
Arcuate nucleus	Arc	Arc
Catecholamine (CA)-synthesizing Neurons in NTS	n/a	NTSCA
Cholecystokinin	CCK	CCK
Cholecystokinin receptor	CCKR	CCKR
Dopamine receptor 2	D2R	DR
Food Intake	n/a	FI
GABA receptor	GABA-R	GABAR
Galanin expressing neurons of LH	n/a	LHGal
Galanin receptor	GAL-R	GalR
Glucagon-like peptide 1 (GLP1)-synthesizing neurons in NTS	NTS-PPG	NTSGLP1
Glucagon-like peptide 1 receptor	GLP-1R	GLP1R
Glucose sensitivity measure (receptor)	n/a	GLUR
Growth hormone secretagogue receptor	GHSR	GHSR
Lateral hypothalamus	LH	LH
Leptin receptor (long form)	LepRB	LepRB
Melanocortin concentrating hormone	MCH	MCH
Melanocortin concentrating hormone receptor type 1	MCH-R1	MCHR
Melanocortin receptor 3 (or 4)	MC3R (MC4R)	MC3R (MC4R)
Neuropeptide Y type 1 receptor	Y1R or NPY1R	Y1R
Neurotensin	NT	NT
Neurotensin receptor 1	NTR1	NTR
Nitric oxide synthase-1-expressing GABAergic neurons	n/a	NGABA
Nucleus accumbens	NAc	NAc
Nucleus tractus solitarius	NTS	NTS
Orexin	OX, OXN	OX
Orexin receptor 1	OX1R	OXR
Oxytocin	OXT	OXT
Oxytocin receptor	OXTR	OXTR
Paraventricular hypothalamus	PVH, PVN	PVH
Pro-opiomelanocortin	POMC	POMC
Ventral tegmental area	VTA	VTA

*Standard abbreviations refer to actual neurobiological entities while variable names (in monotype) refer to computer model variables*.

Table 2**Truth table. (A) Each entry represents an experimental manipulation and its observed effect on food-intake. Data are taken from two review articles (Sohn et al., 2013; Sternson and Atasoy, 2014). Increase, Decrease, and No change refer to statistically significant increases, decreases, or no significant change in the measured quantity from experimentally determined baseline levels. db/db and ob/ob are LepRB deficient and leptin deficient transgenic mice, respectively. (B) The entries concern overall effects on food intake or the activity of certain neural subtypes. Data for experiments 26 through 30 are taken from a review article (Sohn et al., 2013). Data for experiments 31 and 32 are taken from (Kong et al., 2010) and (Schick et al., 2003), respectively. Increase, Decrease, and No change refer to statistically significant increases, decreases, or no significant change in the measured quantity from experimentally determined baseline levels. n/a indicates that relevant data for the corresponding entry are not available**.

**Table:** 

**No**.	**Experiment**	**Food-intake**
**(A)**		
1	POMC knockout	Increase
2	MC3R knockout	Increase
3	MC4R knockout	Increase
4	Y1R knockout	Decrease
5	db/db or ob/ob	Increase
6	LepRB deletion in POMC neurons	No change
7	LepRB deletion in AgRP neurons	No change
8	LepRB deletion in POMC and AgRP neurons	No change
9	LepRB deletion in NTS neurons	Increase
10	LepRB deletion in GABAergic neurons	Increase
11	LepRB expressed only in POMC neurons	Increase
12	5HT1B receptor knockout	No change
13	5HT2C receptor knockout	Increase
14	Photo-stimulation of AgRP neurons	Increase
15	Photo-stimulation of AgRP projection to PVH	Increase
16	Photo-stimulation of AgRP projection to PVH and photo-stimulation of PVH	No change
17	Simultaneous photo-stimulation of AgRP neurons and chemo-inhibition of AgRP projection to PVH	Increase
18	Photo-stimulation of AgRP projections to PVH while blocking GABA receptors on PVH	No change
19	Photo-stimulation of AgRP projections to PVH while blocking Y1R receptors on PVH	No change
20	Chemogenetic activation of AgRP neurons while blocking GABA receptors on PVH	Increase
21	Chemogenetic activation of AgRP neurons while blocking Y1R receptors on PVH	Increase
22	Chemo-inhibition of AgRP neurons	Decrease
23	Simultaneous photo-stimulation of AgRP neurons and POMC neurons	Increase
24	Chemo-inhibition of PVH neurons	Increase
25	Photo-stimulation of AgRP projection to LH	Increase

**Table:** 

**No**.	**Experiment**	**Food-intake**	**POMC**	**AgRP**	**OXT**
**(B)**					
26	Leptin administration	Decrease	Excitation	Inhibition	Excitation
27	Ghrelin administration	Increase	Inhibition	Excitation	n/a
28	5HT1B receptor agonist administration	n/a	n/a	Inhibition	n/a
29	5HT2C receptor agonist administration	Decrease	Excitation	n/a	n/a
30	α-MSH administration	Decrease	n/a	n/a	Excitation
31	Glucose administration	No change	n/a	n/a	n/a
32	GLP-1 administration	Decrease	n/a	n/a	n/a

### Neurobiological basis of the model

The model is based mainly on recent findings obtained in rodents using traditional methods as well as more modern ones including optogenetics and chemogenetics. Both model structure and required input/output behavior (i.e., the truth table) are based directly on findings from the literature. The available data is statistical, consisting mainly of statistically significant increases or decreases in experimentally measureable quantities due to experimentally feasible manipulations. Such data indicate which biological entity (neuron, peptide, etc.) significantly influences the activity of which other entity. One example is increased food intake caused by photo-stimulation of the projections of AgRP neurons to PVH (Betley et al., 2013). Another is excitation of POMC neurons by systemic administration of leptin (Williams et al., 2010). Neural subtypes are included in this initial version of the model only if their behavior has been well-described and their connections with the other subtypes involved in food-intake control have been well-established experimentally. In some cases the presence or absence of specific receptors is demonstrated directly (e.g., using immunohistochemistry) but in other cases receptors are inferred on the bases of the effects of agonists and antagonists. A large set of such findings are gathered together in order to set the model diagram and truth table. In the attempt to include as much relevant data as possible, some amount of generalization over rodent species/strain, sex, age, experimental conditions, various peptide modifications, and receptor subtypes was necessary, despite the fact that all of those factors can bear on food-intake control. For that reason, this initial model should be considered as a rough approximation to the actual food-intake control network, but one that takes a broad range of data into account (see also Discussion).

The five substances represented in the model are leptin, ghrelin, CCK, 5HT, and glucose. These substances were chosen simply because they were the ones used in the experiments describing the behavior of the neural subtypes included in the model. Leptin is a hormone produced mainly by adipose tissue and acts on cognate receptors in brain. LepRB is expressed in various brain regions including Arc, LH, VTA, and NTS. Leptin is anorexigenic (i.e., food-intake suppressing). It inhibits food intake through several pathways (Morton et al., 2006; Klok et al., 2007). Ghrelin is a hormone secreted by the ghrelin cells of the gastrointestinal tract but the hypothalamus is also a ghrelin source (Wren et al., 2000). In contrast to leptin, ghrelin is orexigenic (i.e., food-intake promoting). GHSR is expressed in Arc, LH, VTA, and NTS (Klok et al., 2007). Ghrelin activates AgRP neurons directly through GHSRs and inhibits POMC neurons indirectly through activation of AgRP neurons which in turn inhibit POMC neurons (Cowley et al., 2003). CCK is a hormone produced by the L-cells of the duodenum. Along with its peripheral effects, CCK also acts on cognate receptors in brain, mainly in NTS, to terminate a meal (Peikin, 1989). Serotonin (or 5HT) is a neurotransmitter produced by and secreted from raphe nucleus neurons. Serotonin suppresses food intake through excitation of POMC neurons and inhibition of AgRP neurons by acting on 5HT2CRs and 5HT1BRs, respectively (Tecott et al., 1995; Bouwknecht et al., 2001; Heisler et al., 2006; Xu et al., 2008, 2010). Nutrients such as glucose can affect food intake by acting on brain regions that show sensitivity to specific nutrient levels (Cota et al., 2006; Gillum et al., 2008; Domingos et al., 2013; Sheng et al., 2014).

The arcuate nucleus of the hypothalamus has been the main focus of food intake research over the past decade. Three Arc subtypes are represented in the model: POMC, AgRP, and NGABA. POMC neurons produce α-MSH. Leptin activates POMC neurons, which express LepRB, and POMC neurons activate OXT neurons in PVH, which have MC3Rs and MC4Rs (Balthasar et al., 2005; Hill, 2012). Leptin activation of POMC neurons generally suppresses food intake, which is consistent with the anorexigenic effect of leptin, their main regulator. In contrast to POMC neurons, activation of AgRP/NPY expressing neurons in Arc generally promotes food intake. Distinct subtypes of AgRP neurons express leptin and ghrelin receptors, and leptin and ghrelin inhibit and excite AgRP neurons, respectively (Kohno and Yada, 2012). AgRP is an inverse agonist of MC3/4Rs, and AgRP neurons inhibit OXT neurons in the PVH by blocking MC4Rs (mainly) and MC3Rs (Atasoy et al., 2012). Also AgRP neurons co-express NPY and GABA and inhibit POMC neurons via Y1Rs and GABARs on POMC neurons. Interestingly, deletion of LepRB on AgRP or POMC neurons or both only modestly affects food intake, but deletion of leptin receptors on GABAergic neurons in the hypothalamus of mice mimics the food-intake enhancing effects of whole-body leptin-receptor knockout (Vong et al., 2011). This indicates the important role of GABAergic leptin-receptor expressing neurons in hypothalamus, specifically the GABAergic, nitric oxide synthase-1-expressing (NGABA) neurons in Arc, which inhibit POMC neurons (Leshan et al., 2012). Both AgRP and POMC neurons project to the OX neurons of LH and respectively excite and inhibit them (Elias et al., 1999). AgRP neurons inhibit NAc neurons through the action of NPY on Y1Rs (Van Den Heuvel et al., 2015). NPY reduces the activity of neurons throughout the NAc, affecting the motivation to obtain food (Van Den Heuvel et al., 2015). Ablation of 5HT1BRs on AgRP neurons causes increased food intake (Bouwknecht et al., 2001).

Interest has shifted back to the VMH in recent years (King, 2006) but the connections between VMH neurons and the other neural subtypes mediating food-intake control have yet to be characterized. For that reason we exclude VMH from this version of the model. Beside Arc, the other hypothalamic nuclei we include in the model are PVH and LH. The neural subtype we represent in PVH is OXT. OXT-expressing neurons in PVH express MC4Rs (and MC3Rs to a lesser extent) and are highly innervated by AgRP and POMC neurons (Shah et al., 2014). OXT projections to NTS increase the sensitivity of NTS neurons to peripheral satiety signals such as CCK (Blevins et al., 2003, 2004, 2009; Hill, 2012). OXT neurons also express MCHRs and are inhibited by projections from LH MCH neurons (Parkes and Vale, 1993; Hawes et al., 2000; Chee et al., 2013).

We represent four LH neural subtypes: OX, LHGal, MCH, and NT. OX-expressing neurons in LH are inhibited by glucose and activated by ghrelin (Williams et al., 2008; Perello et al., 2010; Cone et al., 2014). OX neurons project to VTA and activate VTA neurons. This causes increased dopamine release from VTA to NAc and decreased activity of NAc neurons, which is associated with increased food intake (Harris et al., 2005; Choi et al., 2010; Sheng et al., 2014). OX neuron activity is also suppressed by LepRB-expressing neurons in LH. This inhibition is GABA independent and mediated by galanin neuropeptide due to galanin-expressing LH neurons (LHGal) (Laque et al., 2013). OX neurons also project to NTS and reverse the food-intake suppression mediated in this brain region by reversing the stimulatory effect of CCK on NTS neurons, specifically onto catecholaminergic NTS (NTSCA) neurons (Asakawa et al., 2002; Burdyga et al., 2003; Parise et al., 2011).

OX and MCH are the two LH neural subtypes whose activity is modulated by glucose. Unlike OX neurons, which are inhibited by glucose, MCH neurons are excited by it (Kong et al., 2010; Domingos et al., 2013). MCH neurons promote food intake by inhibiting OXT and NAc neurons (Alon and Friedman, 2006; Sears et al., 2010). Unlike OX and MCH neurons, the other two LH subtypes, LHGal and NT neurons, have LepRBs and are activated by leptin (Leinninger et al., 2009; Laque et al., 2013; Goforth et al., 2014). LHGal neurons inhibit OX neurons in LH (Laque et al., 2013), while NT neurons in LH activate VTA neurons via neurotensin secretion (Patterson et al., 2015). The MCH, LHGal, and NT LH subtypes are GABAergic (Leinninger et al., 2009, 2011; Jego et al., 2013).

We represent two neural subtypes in NTS: NTSCA and NTS-PPG (or NTSGLP1, glucagon-like peptide 1-synthesizing neurons in NTS). NTSCA neurons are activated by peripheral signals including CCK (Appleyard et al., 2007). Leptin activates both NTSCA and NTSGLP1 neurons: leptin activates NTSGLP1 neurons directly (Hisadome et al., 2010) and activates NTSCA neurons both by increasing their CCK sensitivity and by activating OXT neurons (via POMC neurons), which further increases their CCK sensitivity (Blevins et al., 2004; Peters et al., 2008; Ong et al., 2015). NTSGLP1 neurons are activated by CCK indirectly through activation of NTSCA neurons (Hayes et al., 2009; Hisadome et al., 2011). Ghrelin decreases the responsiveness of NTSCA neurons to satiety signals (Cui et al., 2011), and GLP1-induced reduction of food intake is suppressed by ghrelin (Chelikani et al., 2006). Activation of NTSCA or NTSGLP1 neurons suppresses feeding (Hayes et al., 2010; Kanoski et al., 2012). NTSGLP1 neurons decrease food reward by exciting NAc neurons and inhibiting VTA neurons (Dossat et al., 2011; Alhadeff et al., 2012; Dickson et al., 2012; Richard et al., 2015).

Along with NTS, VTA and NAc form the non-hypothalamic, output stage of the model (see also next subsection). Peripheral leptin administration inhibits dopamine neurons in VTA and causes acute inhibition of food intake (Hommel et al., 2006; Thompson and Borgland, 2013). GHSR is expressed in VTA, and ghrelin administration increases VTA neuronal activity and dopamine release into NAc (Abizaid et al., 2006; Jerlhag et al., 2006; Jerlhag, 2008). LH NT neurons increase the activity of VTA neurons (Legault et al., 2002; Patterson et al., 2015). An NT receptor 1 (NTR1) antagonist attenuates the rewarding effects of LH activation of VTA (Kempadoo et al., 2013). The NAc (specifically the NAc shell) is involved in food-intake control, and inhibition of neurons in NAc shell increases feeding (Stratford et al., 1998; Zheng et al., 2003).

The data used in the truth table (Table 2) are derived from experiments showing changes in food intake, or in the responses of specific neurons in the food-intake control network, that were due to transgenic, optogenetic, chemogenetic, hormonal, or pharmacological manipulations. Statistically significant changes in food intake are represented as an increase or decrease, and in neuronal activity as an excitation or inhibition. In forming Table 2 we used data that had already been compiled in two recent review articles (Sohn et al., 2013; Sternson and Atasoy, 2014), along with data from a few primary sources (Schick et al., 2003; Kong et al., 2010). Together these articles provide a set of 32 input and observed output pairs that provide a thorough characterization of the input/output behavior of the food-intake control network as it is presently understood. Agreement between the model and the truth table therefore signifies that the model constitutes a valid representation of the available data on the food-intake control network.

### Computational representation and analysis

The food-intake control model takes the form of a feedforward neural network (see Figure 1). In a conventional neural network model, each presynaptic unit is thought to release a single transmitter from its synaptic connections to postsynaptic units, and each postsynaptic unit has a dedicated weight for each synapse from presynaptic units. The food-intake control neural network model is unconventional in that each unit can release more than one neurotransmitter and can have receptors for more than one neurotransmitter and/or other substance. It is also unconventional in that a synapse on one of its units is presynaptically modulated by the other inputs to that unit. The elements of the model are 12 units, representing neurons of specific subtypes in the food-intake control network, and 5 naturally occurring substances (hormones, 5HT, and glucose) that affect those neurons. The response of any unit is a real (floating point) number representing neuronal firing rate, and the amount of any specific transmitter released by a unit is equal to the response of the unit. The response of any unit is a function of its net input from other elements (substances and/or units) plus its own intrinsic bias. Computation of the net input to a unit is somewhat unusual in this unconventional network.

The net input to any unit is determined by the amount of all substances it receives and by the strengths of its receptors for each substance. For any unit, the amounts of any specific substance from all sources are summed, and the sums for each specific substance are then weighted by the strength of the unit's receptor for that substance (i.e., cognate receptor for that specific substance). In order for the model to reproduce the data represented in the truth table (Table 2), the units also had to respond to drugs that could modulate receptor strengths or act as receptor ligands in addition to the naturally occurring substances. Thus each receptor in each unit was activated according to the total amount of its cognate ligands (sum of all substances and drugs activating that receptor) and any modulation of receptor strength due to drugs (or in one case due to neurotransmitters and hormones). The net input to any unit was then the sum of all its receptor activations plus its intrinsic bias. Receptors can make either an excitatory or an inhibitory contribution to net input. Since real neurons cannot have negative firing rates the response of any unit is equal to its net input bounded at zero. There is no upper bound and the maximal responses of the units varied (see Supplementary Material). Because the focus of this analysis is on response patterns (i.e., the responses of the units relative to one another), unit responses are expressed as percentages of their maximal responses in figures and tables.

Unit responses and network interactions are represented in computer programs written in two different programming languages, one declarative and the other imperative. The declarative language we use is Maude (Clavel et al., 2007). All units, receptors, substances, and drugs are represented in Maude as operators having certain attributes. For example, in cell(POMC, Net, Ne) the operator cell is used to assign the floating-point number held in variable Ne to the Net input to the POMC unit (note that all computer variables and code are rendered in monotype font). Similarly, in rec(POMC,LepRB,R2), the operator rec is used to assign the floating-point number held in variable R2 to the strength of LepRB of the POMC unit. As a declarative language, all statements in Maude are declarations. The set of Maude declarations that determine the response of POMC, and the levels of the transmitters it releases, is shown below.


crl do(1) cell(POMC, Bias, Bi) rec(POMC,
   LepRB, R2)
   rec(POMC, Y1R, R3) rec(POMC, GABAR, R4)
   rec(POMC, FHT2CR, R5)
   Leptin(X2) tran(AgRP, NPY, T1)
   tran(AgRP, GABA, T2)
   tran(NGABA, GABA, T3) FHT(X3)
   risperidone(X4) FHT2Cag(X5)
   cell(POMC, Net, Ne) = >
   do(8) cell(POMC, Bias, Bi) rec(POMC,
   LepRB, R2)
   rec(POMC, Y1R, R3) rec(POMC, GABAR, R4)
   rec(POMC, FHT2CR, R5)
   Leptin(X2) tran(AgRP, NPY, T1)
   tran(AgRP, GABA, T2)
   tran(NGABA, GABA, T3) FHT(X3)
   risperidone(X4) FHT2Cag(X5)
   cell(POMC, Net, ((R2 ^*^ X2) - (R3 ^*^ T1) -
   (R4 ^*^ (T2 + T3)) +
   ((R5 - (X4 ^*^ 0.5)) ^*^ (X3 + X5)) + Bi))
   if Ne =/= ((R2 ^*^ X2) – (R3 ^*^ T1) –
(R4 ^*^ (T2 + T3)) +
   ((R5 - (X4 ^*^ 0.5)) ^*^ (X3 + X5)) + Bi) .

eq do(8) cell(POMC, Live, Li) cell(POMC,
   Net, Ne)
   cell(POMC, Res, Re) =
   do(9) cell(POMC, Live, Li) cell(POMC,
   Net, Ne)
   cell(POMC, Res, Li ^*^ max(0.0, Ne)) .

eq do(9) cell(POMC, Res, Re) tran(POMC,
   aMSH, T1) =
   do(100) cell(POMC, Res, Re) tran(POMC,
   aMSH, Re) .


Declarations in Maude can be understood as term rewrites in which a term matching the left-hand side of an assignment symbol (= or =>) is replaced with the term matching the right-hand side. The first declaration in the POMC set computes the Net input to POMC from the levels of the substances it receives from other elements, the strengths of its cognate receptors, and its Bias. Note that this declaration is conditional and executes only if the current Net input to POMC is changed in so doing. This declaration illustrates how GABA from two sources, the AgRP and the NGABA units (variables T2 and T3), are summed and how the sum is weighted by the strength of the POMC unit's GABAR (variable R4). It also illustrates how the strength of the POMC unit's FHT2CR (variable R5) is modulated by the drug risperidone (variable X4) before it is activated by endogenous FHT and the 5HT2CR agonist drug FHT2Cag (variables X3 and X5). The second declaration computes the POMC unit's response (attribute Res) as its Net input (variable Ne) bounded at 0 and then multiplied by the value of its Live attribute (variable Li), which is 1 if the unit is alive and 0 if it has been lesioned or completely inactivated. The third declaration simply sets the level of the transmitter aMSH released by the POMC unit to its response (variable Re). These three declarations together determine the POMC unit's response and the level of the single transmitter it releases. Similar sets of declarations determine responses and transmitter levels for the other units.

In addition to being conditional, the first declaration in the POMC set differs in a critical way from the other two because the first declaration is a rule (specifically a conditional rule, crl, with assignment symbol = >) while the other two declarations are equations (specifically non-conditional equations, eq, with assignment symbol =). Rules differ from equations in Maude because an equation must execute whenever it applies but an applicable rule may execute or not. For the POMC set, the first declaration, as a rule, may execute or not but when it does it rewrites the do operator from do(1) to do(8). This makes the second declaration applicable and so, as an equation, it must execute and when it does it rewrites do(8) to do(9). That makes the third declaration applicable and so, as an equation, it also must execute and when it does it rewrites do(9) to do(100).

This update strategy for POMC is also used in the sets of declarations that update the other hypothalamic units: AgRP, NGABA, OXT, MCH, OX, LHGal, and NT. The first declaration in any hypothalamic-unit set *can* (but does not necessarily have to) execute with do(1) and it rewrites the do operator to the value appropriate to the next declaration (an equation) in its set. The last equation in any hypothalamic-unit set rewrites the do operator to do(100). The declarations that determine the level of food intake (element FI) and of the non-hypothalamic units that immediately influence it (NTSGLP1, NTSCA, NAc, and VTA) are all expressed as equations. Among the non-hypothalamic units NTSCA is somewhat special in that the strength of its CCKR is modulated by Leptin, Ghrelin, OX, and OXT as follows. The net input due to Leptin, Ghrelin, OX, and OXT is calculated as the sum of the level of each substance multiplied by the strength of its cognate receptor on NTSCA. If this sum is greater than zero, and if both CCK and CCKR are greater than zero, then the net input to NTSCA is calculated as the sum of the level of each of Leptin, Ghrelin, OX, OXT, *and* CCK multiplied by the strength of its cognate receptor on NTSCA. The sequence of declarations that updates these four non-hypothalamic units and FI is initiated with do(100). In this way whenever one of the hypothalamic units updates, any effect of that update on the responses of the non-hypothalamic units and on FI must be registered immediately. After FI updates it resets do to do(1), enabling applicable rules (for the hypothalamic units) to execute (or not) once again.

The distinction between rules and equations has profound implications for the way in which transitions occur in the state of a model specified in Maude. Specifically, only rules can cause state transitions. Equations can elaborate the state but cannot change it. Given our modeling strategy, the state will transition with every update of a hypothalamic unit (AgRP, POMC, NGABA, OXT, MCH, OX, LHGal, or NT). Since a state in the model corresponds to a response configuration, the only states we consider are those involving differing patterns of responses among the hypothalamic units. Crucially, the only response configurations we consider are those that result because a hypothalamic unit that can update has not yet updated. This is a limited set of response configurations but it is parsimonious, since it involves interruptions in known interactions that would otherwise take place. They correspond to limitations in the responses of specific neural subtypes that plausibly could be imposed by neurons external to the food-intake control system. Our modeling strategy, in which hypothalamic-unit updates are rules but non-hypothalamic-unit updates are equations, ensures that any changes in food intake will be due only to changes in the configuration of the responses of the hypothalamic units (AgRP, POMC, NGABA, OXT, MCH, OX, LHGal, or NT), and not due to failure to update of any of the non-hypothalamic units (see also Discussion).

The beauty of rules, which unlike equations can execute or not, is that Maude can execute the rules in a model in all possible orders. Specifically, from any initial state Maude executes all applicable equations. From that elaborated initial state she executes each applicable rule, and then executes any equations made applicable by that rule. This begins the process of enumeration of the state transition tree, where the initial state is the root, and each rule execution is a branch to the first layer of the tree. Then, from each layer 1 state, Maude again executes each applicable rule and executes any equations made applicable by that rule, thus branching out further and forming the states at layer 2 of the tree. The breadth of the tree grows geometrically with each layer, and the growth rate depends on the number of rules that are applicable in each state. All the rules in the model are conditional, and execute only if the net input to a unit will change as a result of that rule, so not all rules are applicable from every state in the model. Still, a tree with up to eight rules (one for each hypothalamic unit) potentially applicable in any state grows rapidly. The power of Maude lies in her ability to search and analyze such a state transition tree.

When given a search command, Maude will form and search the state transition tree for all states that meet a given set of conditions and count them. The following is an example search command.


search init =>+ cell(AgRP, Res, X1) cell
   (POMC, Res, X2) act(FI, Res, X3)
   S:State such that
   X1 == 0.0 and X2 > 25.69 and X3 > 124.14 .


In this command init is shorthand for some initial state, and the two cell operators and the activation (act) operator indicate that the responses of interest are those of AgRP, POMC, and FI. S:State is shorthand for all the other elements whose levels are not conditions of this search. Given this command (with assignment symbol =>+) Maude will search the state transition tree for all states in which the levels of AgRP, POMC, and FI are equal to 0.0, greater than 25.69, and greater than 124.14, respectively (floating-point numbers have been truncated for clarity). These values correspond to AgRP inactive, POMC active at any level above its baseline, and FI more than 30% greater than its baseline, which is considered as increased food intake (see below). For the Average model parameterization (see Results), Maude found six such states. Each state satisfies the same conditions but does so in its own way and, importantly, with its own unique pattern of unit responses. Characterization of all response configurations (patterns of responses of the units in the network) that are consistent with specific conditions is central to the analysis presented here.

In the above search command the assignment symbol =>+ means that Maude should search for any state achieved after one or more rule executions. In contrast the command


search init = >! S:State .


with assignment symbol =>! means that Maude should search for all terminal states. Terminal states are those in which no further rules are applicable. Thus, each branch of the state transition tree ends in a terminal state. In general, models are not guaranteed to have terminal states. As a feedforward network, the food-intake control model terminated along all branches of the state-transition tree. Moreover, from a specific initial state, the terminal state ultimately reached at the end of every branch of the tree was the same state. This means that unit response updates will reach the point where further updates would not change the responses of any units, and at that point the pattern of unit responses (i.e., the network response configuration) will be the same for any given initial state no matter the order of updates of the units in the network. The normal initial state of the model is characterized by a level of endogenous substances (Leptin, Ghrelin, CCK, FHT, and Glucose) that produces a medium, baseline level of FI. The (single) terminal state reached from the normal initial state defines the baseline responses of all units. This terminal state was reached along all branches of the state-transition tree by layer 9.

An important aspect of this analysis is that differences in network response configuration occur only in non-terminal states (there are many of these). A non-terminal state is a state in which a unit could update but has not yet updated. The key assumption of this analysis is that non-terminal states in the model correspond to possible response configurations of the real food-intake control network that result when neurons outside the network limit the responses of the neurons inside the network, as presently construed. Thus, the analysis explores a restricted subset of possible modulations of the food-intake control network, specifically those that result from limitations in the responses of the well-described hypothalamic neural subtypes that are known to compose it. Even with this parsimonious restriction, the analysis still identifies many different response configurations that are nevertheless associated with similar levels of food intake, and suggest that incongruous findings can be reconciled by viewing them within the larger context of the whole network.

Maude allows us not only to search the state transition tree for states that satisfy certain conditions but also allow us to determine temporal relationships between states that are invariant in that they are independent of the order of rule executions. In declarative environments such as Maude this is done using temporal-logic model-checking. The following is an example of a model-check command.


modelCheck(FIM(init), AgRPeqZero /\
   POMCgtBL = > FILow) .


In this command FIM is an operator that wraps the entire state of the food-intake control model, AgRPeqZero is the property that AgRP equals zero, POMCgtBL is the property that POMC is greater than its baseline, and FILow is the property that FI is low (see Results). The symbols /\ and = > stand for the logical connectives “and” and “implies,” respectively. We make extensive use of the “implies” logical connective in our analysis. The above command using “implies” asks Maude to check whether FI is low *whenever* AgRP is zero and POMC is greater than its baseline. This statement is false, meaning that there is at least one state in which AgRP is zero and POMC is greater than its baseline but FI is not low, and it is false in all model parameterizations examined (see Results). We use temporal-logic analysis to show precisely in what ways the incongruous findings differ from the expected findings, and this allows us to generate experimentally testable predictions concerning the food-intake control network.

The same unit responses and network interactions represented in Maude, a declarative language, are also represented in MATLAB™, an imperative language. This is done to provide a crosscheck, and to leverage the separate strengths of the two programming modalities: Maude is used for state-space search and temporal-logic model-checking while MATLAB is used for model parameter optimizations. In MATLAB all model units are represented as structures having different fields. For example, the net input to POMC is POMC.Net, while the strength of LepRB on the POMC unit is POMC.LepRB. The set of MATLAB commands that determine the response of POMC, and the levels of the transmitters it releases, is shown below.


POMC.Net = POMC.Bias + POMC.LepRB ^*^ Leptin +
   −POMC.GABAR *(AgRP.GABA + NGABA.GABA) +
   -POMC.Y1R * AgRP.NPY +
   (max(0, POMC.FHT2CR - (risperidone/2))) *
   (FHT + FHT2Cag);

POMC.Res = max(0, POMC.Live * POMC.Net);

POMC.aMSH = POMC.Res;


This update strategy for POMC is also used in the sets of commands that update all the other units in the MATLAB version of the model. The statements in the MATLAB and Maude version implement exactly the same computations but in different ways. Unlike declarative programs (such as those written in Maude) statements in imperative programs (such as those written in MATLAB) are commands that execute in the order in which they are listed in the program. In the MATLAB version, all of the commands are placed within a for-loop that runs for a number of iterations sufficient to ensure that all units cannot undergo further update. From all initial states tested the Maude and MATLAB versions reach exactly the same terminal states. The crosscheck indicates that the reported results are unlikely to be corrupted by programming error.

The parameters of the model are the strengths of the receptors and the biases of each unit. These parameters number 51 in all. The MATLAB version was used to optimize these parameters using the genetic algorithm (GA) as implemented in MATLAB. Specifically, the GA adjusted parameters so as to minimize the difference between observed and simulated behavior over the entire truth table, which represents the results of 32 actual experiments (see Table 2). As appropriate to the statistical nature of the available data, the truth table indicates statistically significant changes in food intake or neuronal activity rather than absolute quantities. For the purposes of parameter optimization, a simulated change in food intake or unit activity counts as a significant change when it is more than 30% up or down from its baseline level. When a simulated change agrees with an observed change the error for that truth-table entry is zero. We ran the GA 1000 times with a population size of 1000 that evolved for 5100 generations (following the MATLAB GA recommendation that the number of generations should equal 100 times the number of parameters). Most of the GA runs (about 96%) did not achieve zero error over the entire truth-table after 5100 generations. For selected parameter sets that did achieve zero error overall, the 30% criterion produced simulated changes that were significantly different from baseline, with a *t*-test *p*-value at or below that of the experimental data in the truth table (see Results).

## Results

The results are generated from computational analysis of a model of the interactions between the neural subtypes known to contribute to food-intake control. The overall goal of the analysis is to explore a subset of the response configurations of the model in order to find configurations that are compatible with recent, unexpected findings. The subset of model configurations considered are only those that result from limitations in the responses of the neural subtypes that would otherwise occur given the interactions as represented in the model (see section Computational Representation and Analysis). The initial model takes a broad range of data into account but, of necessity, can only approximate the real food-intake control network (see section Neurobiological Basis of the Model), so the subset of model configurations must be considered as a subset of the subset of possible configurations of the real food-intake control network. The main assumption is that if the model subset includes configurations that are compatible with unexpected findings, then the real network superset must include those configurations also. Likewise, any response patterns that distinguish expected from unexpected model configurations should distinguish real configurations also. The main predictions of the model are that these distinguishing response patterns should be exhibited by the real food-intake control network, and their existence could help explain how the anomalous findings could arise.

The model is instantiated in computer programs written in two different programming languages, both to provide a crosscheck and to leverage the separate strengths of two complementary programming modalities. The programming languages are Maude, a declarative language used for state-space search and temporal-logic analysis of the model, and MATLAB, an imperative language used for computationally intensive model parameter optimizations (see section Computational Representation and Analysis). The goal of model parameter optimization (using MATLAB) was to find a set of parameters (receptor strengths and unit biases) that minimized the error between simulated and actual changes in food intake over a corpus of experimental observations (i.e., the truth table; see Table 2). Repeated optimizations revealed many different sets of parameters that all achieved zero error over the truth table. Model analysis proceeded using several different parameter sets, and they all produced similar results (see sections Model Analysis: Focus on AgRP and POMC Neurons in Arc, and Model Analysis: Focus on GABAergic Neurons in LH). One goal of model analysis (using Maude) was to determine the numbers of total system states (network response configurations) that were compatible with specific experimental findings. These results showed that the same response pattern among a subset of units could be associated with different levels of food intake and, conversely, that the same change in food intake could be associated with multiple network response configurations. These results provide potential resolutions to important apparent inconsistencies in the current data set on the neural control of food intake. Another goal of model analysis was to find invariant temporal relationships between unit response patterns in order to derive predictions that could be tested experimentally. The results are detailed throughout the remainder of this section.

### Model parameter optimizations

The model takes the form of a network of interconnected neural elements (units). The response of any unit is the sum of all its receptor activations, where each receptor activation is computed as the product of the receptor strength and the sum of the levels of the cognate ligands it receives from other sources (endogenous hormones, glucose, transmitters, drugs, etc.). Each unit also has a bias that is added to the summed receptor activations, and the total is bounded at zero (negative responses are disallowed). The parameters of the model, numbering 51 in all, are these response strengths and unit biases (the complete list of parameters is provided in Table S1 of Supplementary Material). The 51 parameters are optimized so as to minimize the error over the truth table (Table 2; see also section Neurobiological Basis of the Model) using the GA implemented in MATLAB (see also section Computational Representation and Analysis).

The truth table is organized in terms of experimental manipulations of the food-intake control system and the resulting, observed changes in food intake or neural responses. Most of the data for the truth table are taken from two review articles (Sohn et al., 2013; Sternson and Atasoy, 2014) (see also section Neurobiological Basis of the Model). Because the data are derived from various labs under differing conditions, the results are expressed in terms of statistically significant increases or decreases in food intake or neural responses, rather than in terms of absolute quantities. The truth table is separated into two parts. The first part (Table 2A) relates experimental manipulations such as total or cell-specific knockouts of hormones or receptors, or opto/chemogenetic activation or suppression of specific neural subtypes, to statistically significant increases or decreases, or to no significant change, in food intake. The second part of the truth table (Table 2B) relates experimental administration of hormones, drugs, or nutrient (i.e., glucose) to statistically significant changes in food intake or in the activities of specific neural subtypes (i.e., POMC, AgRP, and OXT neurons). The error function (inverse fitness function) for the GA assigns zero error to any experiment/result truth-table entry when the simulated change in food intake or unit activity matches the observed change. A simulated change in food intake or unit activity counts as an increase or decrease when it is more than 30% up or down from the baseline level (i.e., the level an element reaches under baseline conditions; see section Computational Representation and Analysis). Changes less than 30% count as no change.

**Figure 2:**
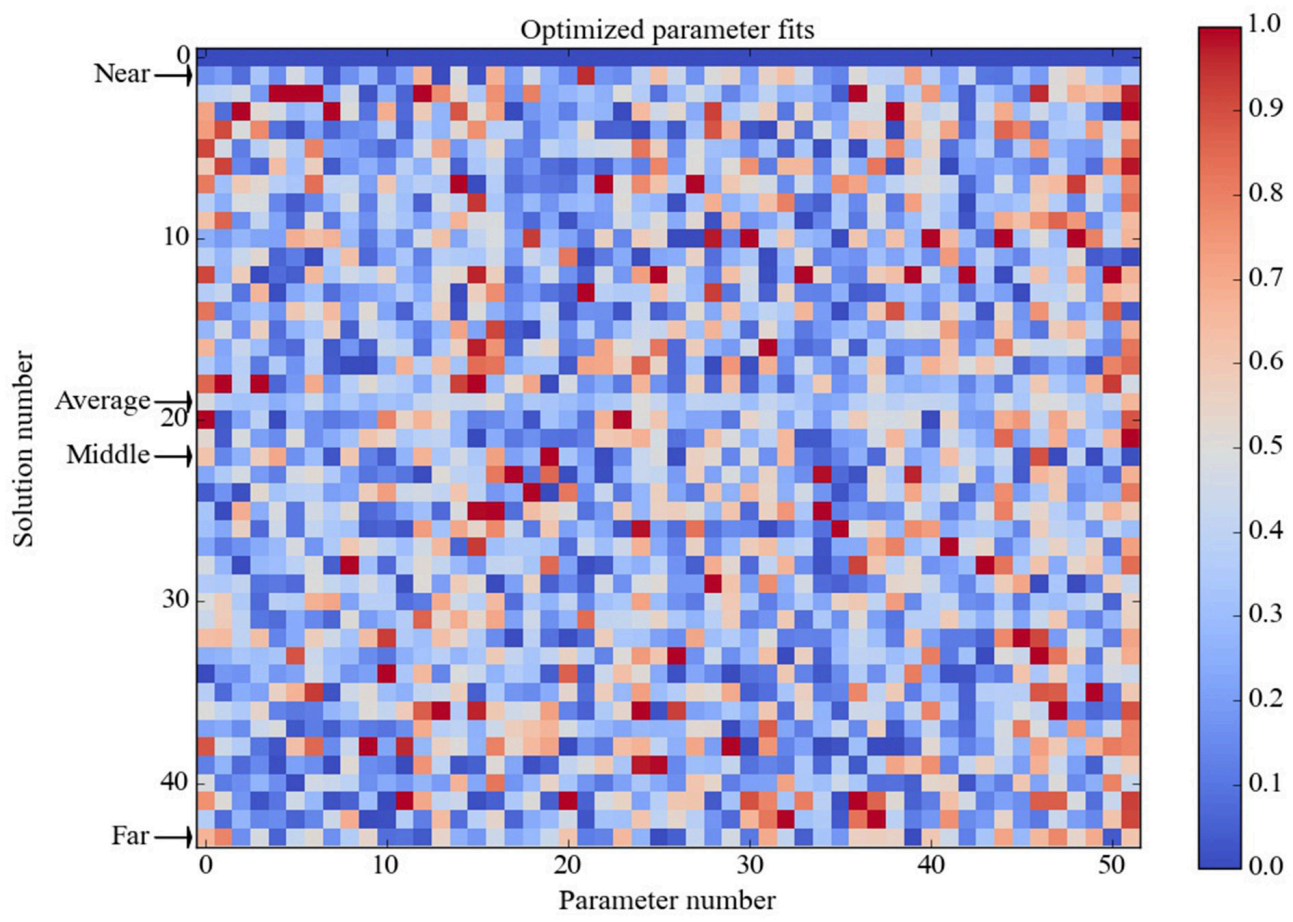
**Heat map of optimized parameter vectors**. Each of the 42 solutions is a vector of model parameter values, optimized to produce perfect correspondence between model and observed responses (zero error over the truth table). Solution vectors are normalized in [0, 1] and ordered by Euclidian distance from the zero vector (simply a vector of 51 zeros), which is shown in the first row. Analysis was conducted on the model separately parameterized with each of the four solution vectors as indicated: Near is nearest the zero vector, Far is farthest from it, Middle is in between, and Average is the element-wise mean of all 42 solution vectors (which is itself a solution vector). The Average solution vector was used to generate the data shown in **Figures 4–7**; figures generated using the other three solution vectors are shown in Supplementary Material. Analysis showed that the model had the same basic behavior for all four of these solution vectors.

Using this 30% criterion, the model only needed to produce food-intake and unit-response values that fell within specific ranges relative to baseline levels. Different model parameterizations (i.e., specific values for receptor strengths and unit biases) could produce different absolute food-intake and unit-response values and still achieve zero error over the entire truth table, and so it was not surprising to find that many different model parameterizations achieved zero overall error. Out of 1000 runs the GA found 42 parameter vectors that achieved zero error over the truth table. The vector of the element-wise averages of these 42 parameter vectors also achieved zero error over the truth table. To provide a qualitative assessment of possible clustering of these optimized parameter vectors they are ordered based on their Euclidian distance from the zero-vector (simply a vector of 51 zeros) and displayed in Figure 2. The 42 optimized parameter vectors do not appear to cluster based on Euclidian distance, and cluster analysis using cophenetic correlation or K-means clustering (not shown) did not reveal obvious clustering either. Using the ordering by Euclidian distance (Figure 2), we chose four parameter vectors for further analysis. They are the vector nearest to the zero-vector (Near), furthest from the zero-vector (Far), and the vector in the middle of the range (Middle), as well as the average vector over all 42 optimized parameter vectors (Average).

**Figure 3:**
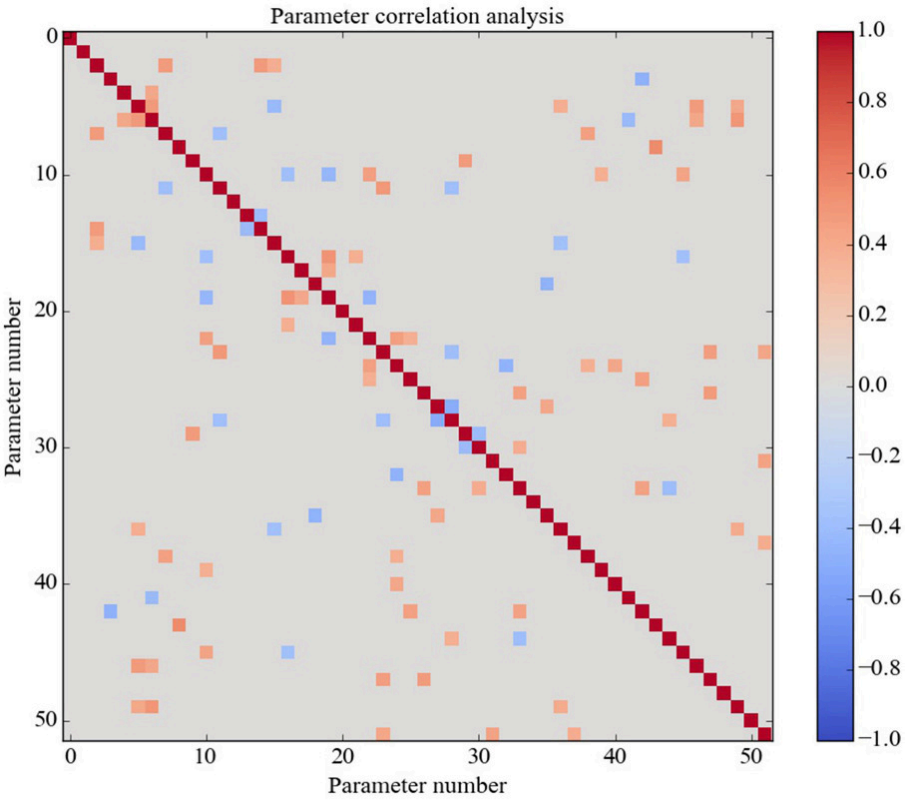
**Correlations between the parameters over all optimized solutions**. There are 51 parameters in all. Non-significant correlation coefficients are shown as zero. Almost all parameters have a significant correlation with some other parameters. Of course, all parameters are perfectly correlated with themselves (red diagonal).

The 42 optimized parameter vectors vary but the GA found zero-error parameter vectors on only 4.2% of runs (42 out of 1000), suggesting that there are many ways to achieve zero error over the truth table but those ways are specific to the model. To get a view on this specificity, the pairwise correlations among all 51 model parameters were calculated and are shown in Figure 3. Only statistically significant correlations (*p* < 0.01) are shown. The correlation analysis shows that most of the parameters are correlated with some other parameters either positively or negatively. This analysis suggests that there are many ways to solve the optimization problem because there are many ways to achieve the needed correlations between the parameters.

A statistical test indicates that the various parameterizations produced changes in food intake (and in some neural responses) that are consistent with the data in the truth table. The neural activity changes in the truth table are few in comparison to the 31 listed changes in food intake (Tables 2A,B), so we focused on simulated changes in food intake to assess the statistical correspondence of our model with the data. Specifically, for each of the four parameterizations examined (Near, Far, Middle, and Average), we grouped all of the increased, decreased, and no-change simulated food-intake values into three separate distributions and used the *t*-test to assess the pairwise differences in their means. In all four cases, the model parameterized with connection weights and unit biases optimized using the GA produced differences in food intake significant at the *p* < 0.001 level, which is at or lower than the *p*-value of the data on which the truth table is based. This shows that a simulated response-change criterion of 30% is sufficient for the GA to find parameterizations that allow the model to reproduce the data to the significance level of the reported experimental findings.

The model analysis results presented in the rest of this section were obtained using all four parameter sets (Near, Far, Middle, and Average), but the results shown in the figures were obtained using only the Average parameter set. Figures showing the corresponding results obtained using the other three parameter sets (Near, Far, and Middle) are qualitatively similar and are available in Supplementary Material. The model parameterized with each of the four parameter sets was analyzed using state-space search and temporal-logic model-checking in Maude. The analysis was directed toward the resolution of paradoxes in the literature on the food-intake control network, specifically those concerning hypothalamic AgRP, POMC, and LH GABA neurons, and toward the generation of experimentally testable predictions on the behavior of this real neural network.

### Model analysis: focus on AgRP and POMC neurons in Arc

It is well-established in the food-intake control literature that AgRP neurons in Arc are orexigenic (promote food intake), while POMC neurons in Arc are anorexigenic (suppress food intake). Supporting this view are findings showing that leptin, an anorexigenic hormone, inhibits AgRP but excites POMC neurons (Elias et al., 1999; Cowley et al., 2001), while ghrelin, an orexigenic hormone, excites AgRP neurons that then inhibit POMC neurons (Kamegai et al., 2001; Cowley et al., 2003). Fasting, which decreases leptin but increases ghrelin levels, also increases AgRP but decreases POMC neuron activity (Hahn et al., 1998; Breen et al., 2005). Optogenetic studies reveal that selective ablation or inactivation of AgRP neurons decreases, but activation increases, food intake, while selective ablation or inactivation of POMC neurons increases, but activation decreases, food intake (Cone, 1999; Kalra et al., 1999; Gropp et al., 2005; Morton et al., 2006; Aponte et al., 2011). Together these studies support the general view that the AgRP-high/POMC-low pattern is associated with increased food intake, while the AgRP-low/POMC-high pattern is associated with decreased food intake.

Interestingly, a recent study shows that the well-established relationship between the AgRP/POMC activity pattern and food intake can be completely reversed when animals are exposed to the sensory qualities of food. Using fiber photometry, Chen et al. (2015) show that exposing mice to food-odor stimuli, which activates feeding, can also inhibit AgRP but excite POMC neurons (AgRP-low/POMC-high), which is opposite to the expected orexigenic pattern. The response is more pronounced in fasted animals, but if the food is particularly palatable (e.g., peanut butter) then exposure to it can activate feeding and evoke the AgRP-low/POMC-high pattern even in sated mice. Most intriguingly, when mice exposed to actual food were allowed to feed, the unexpected AgRP-low/POMC-high activity pattern persisted right up until food consumption began. Taken together, the available data show clearly that the AgRP-low/POMC-high activity pattern can be associated either with decreased (expected) or increased (unexpected) food intake. This is the AgRP/POMC paradox.

**Figure 4:**
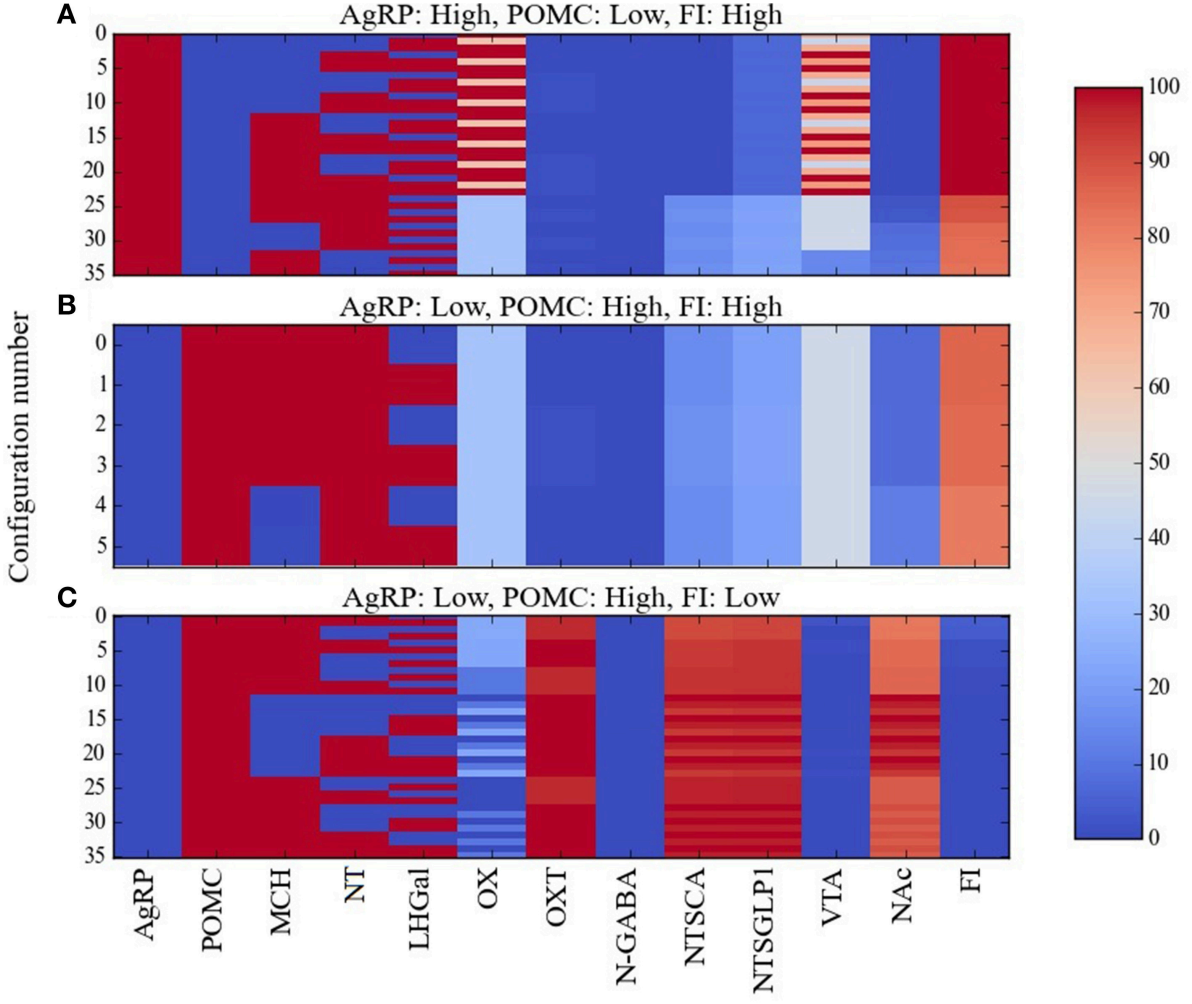
**Percent activity of network units for different patterns of AgRP and POMC activity**. Response configurations corresponding to high food intake are shown when **(A)** AgRP is active and POMC is inactive, or **(B)** when AgRP is inactive and POMC is active. Response configurations corresponding to low food intake are shown when **(C)** AgRP is inactive and POMC is active. Food-intake levels at or above 81% are considered high while those at or below 43% are considered low (corresponding to break points in the FI range). Note that the AgRP/POMC/FI patterns in **(A,C)** are expected but the pattern in **(B)** is anomalous (unexpected, paradoxical).

A resolution to the AgRP/POMC paradox could be found by considering that AgRP and POMC neurons do not act alone but together with other neurons in a larger network, and that response configurations (i.e., overall patterns of the responses of the neurons in the network) exist in which the pattern AgRP-low/POMC-high occurs along with high food intake. In our attempt to resolve this paradox we used Maude to search the model for unit response configurations in which the two different AgRP/POMC response patterns could be associated either with high or low food intake. Many configurations were compatible with the expected AgRP-high/POMC-low pattern and high food intake. Specifically, there were 36, 40, 40, and 46 configurations in the Average, Near, Middle, and Far parameter cases that had the expected AgRP-high/POMC-low/FI-high pattern. Response configurations for the Average case with AgRP-high/POMC-low/FI-high are shown as a heat map in Figure 4A. Response configurations compatible with the paradoxical AgRP-low/POMC-high pattern and high food intake existed but were fewer. Specifically, there were 6, 16, 6, and 10 configurations in the Average, Near, Middle, and Far parameter cases that had the paradoxical AgRP-low/POMC-high/FI-high pattern. Response configurations for the Average case with AgRP-low/POMC-high/FI-high are shown in Figure 4B. Our interpretation of these modeling results is that there are at least some response configurations in the real food-intake control system in which low AgRP and high POMC neuron activity is associated with high food intake, and those configurations are brought about through modulation by factors not currently represented in the model (see Discussion).

**Figure 5:**
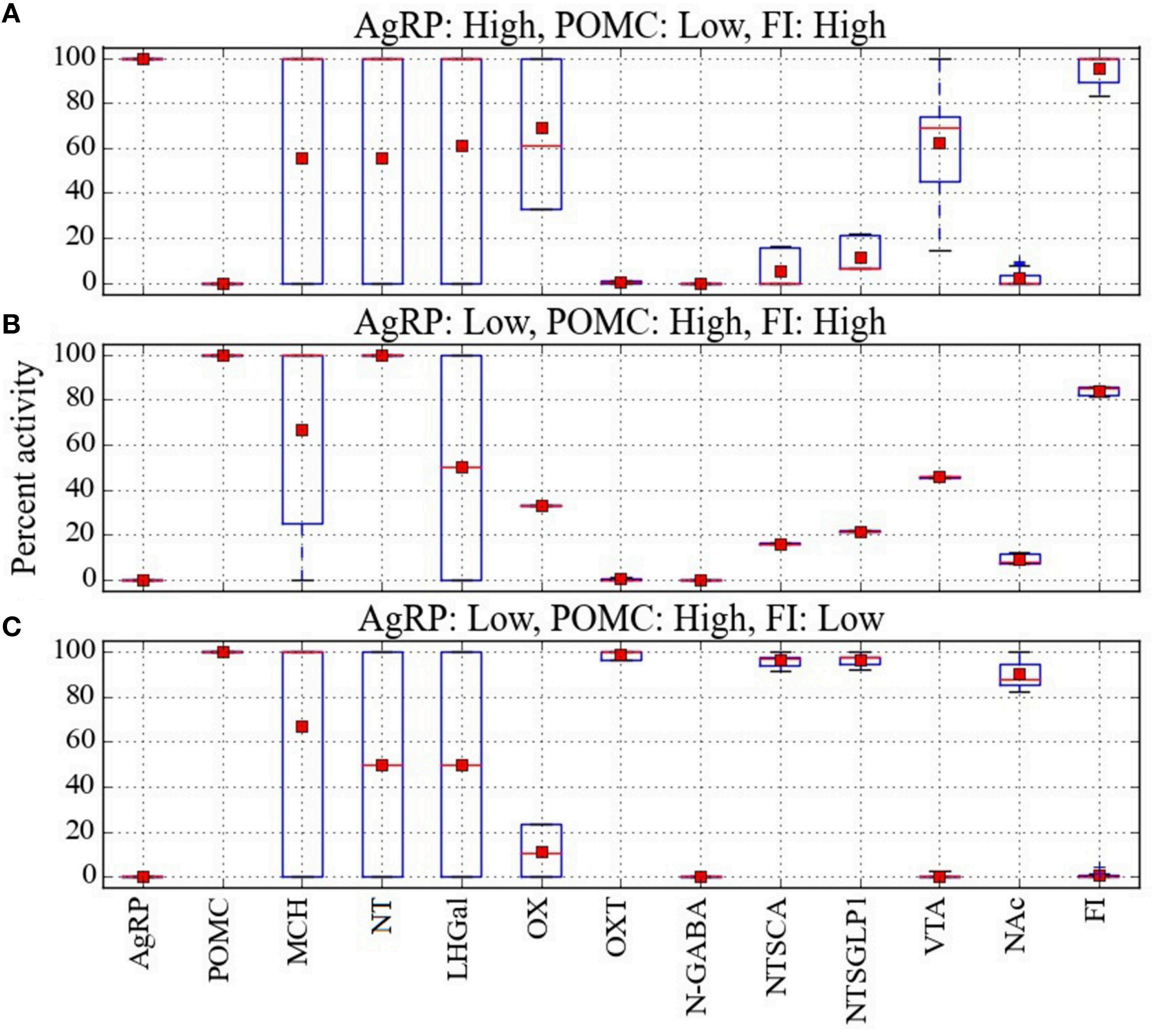
**Analysis of the percent activity for different patterns of AgRP and POMC activity**. Mean activity of each network unit taken over all configurations in cases where **(A)** activation of AgRP and inactivation of POMC is associated with high food intake, **(B)** inactivation of AgRP and activation of POMC is associated with high food intake, and **(C)** inactivation of AgRP and activation of POMC is associated with low food intake. Red squares and lines indicate mean and median, respectively. Blue boxes and bars indicate the interquartile range and the entire range of data, respectively. From the mean values it seems that the anomalous pattern, in which inactivation of AgRP and activation of POMC is associated with high food intake, could occur when OXT is low, OX is midrange, and NT is high.

The model provides a network-level perspective on response configurations compatible with the paradoxical AgRP-low/POMC-high/FI-high pattern, and it allows us to ask in what ways the expected AgRP-high/POMC-low/FI-high and the unexpected AgRP-low/POMC-high/FI-high configurations might differ. For comparison purposes, response configurations compatible with the expected AgRP-low/POMC-high/FI-low pattern are shown in Figure 4C. In comparing the heat maps in Figures 4B,C, it appears that the paradoxical AgRP-low/POMC-high/FI-high pattern can occur when the activity of the OXT unit is zero. To facilitate this comparison, the mean activity of all units in the Average parameter case over all configurations are shown for the AgRP-high/POMC-low/FI-high, AgRP-low/POMC-high/FI-high, and the AgRP-low/POMC-high/FI-low patterns in Figures 5A–C, respectively. These show for the Average parameter case that what distinguishes the paradoxical AgRP-low/POMC-high/FI-high configurations from the expected AgRP-low/POMC-high/FI-low configurations are higher average NT and OX activities and lower average OXT activity for the paradoxical configurations. The OXT difference is especially dramatic (see Figures 5B,C). OXT neurons can be activated by POMC neurons (see section Neurobiological Basis of the Model) and it is possible that a substantial portion of the anorexigenic action of POMC neurons is mediated through OXT neurons. The model suggests that part of the explanation for the paradoxical AgRP-low/POMC-high/FI-high pattern is that it occurs when certain factors prevent POMC activation of OXT neurons (see also Discussion).

Temporal-logic analysis can be used to get a more precise understanding of the conditions that must prevail in order for the model to produce a high level of food intake even when AgRP is inactive and POMC is active. The analysis concerns NT, OX, and OXT, which were the units identified from the state-space search results reported above as potentially permissive of the anomalous AgRP-low/POMC-high/FI-high activity pattern. The results of the temporal-logic analysis for the anomalous AgRP-low/POMC-high/FI-high case are presented in Table 3. All of the logical propositions tested are in the form of implications in which the antecedent involves some conditions on the activity of NT, OX, and OXT (or no condition, NC), while the consequent is high FI (or in one case low FI). In all cases tested AgRP is zero while POMC is active at a level higher than its baseline. The temporal-logic analysis results reported in Table 3 are the same for all model parameterizations: Average, Near, Middle, and Far.

**Table 3:** **Temporal-logic analysis: focus on Arc AgRP and POMC network units**.

**Row**	**Antecedent**	**Consequent**	**Value**
1	NT = NC and OX = NC and OXT = NC	Implies that FI = high	False
2	NT = NC and OX = NC and OXT = NC	Implies that FI = low	False
3	NT> 0 and OX = NC and OXT = NC	Implies that FI = high	False
4	NT = NC and OX> 0 and OXT = NC	Implies that FI = high	False
5	NT = NC and OX = NC and OXT = 0	Implies that FI = high	False
6	NT> 0 and OX> 0 and OXT = NC	Implies that FI = high	False
7	NT = NC and OX> 0 and OXT = 0	Implies that FI = high	False
8	NT> 0 and OX = NC and OXT = 0	Implies that FI = high	False
9	NT> 0 and OX> 0 and OXT = 0	Implies that FI = high	False
10	NT = NC and OX> 20% and OXT = NC	Implies that FI = high	False
11	NT> 0 and OX> 20% OXT = NC	Implies that FI = high	False
12	NT = NC and OX> 20% and OXT = 0	Implies that FI = high	False
13	NT> 0 and OX> 20% and OXT = 0	Implies that FI = high	True

*NC stands for no condition. OX > 20% indicates OX is in the upper 80% of its activity range, OXT < 30% indicates OXT is in the lower 30% of its range, and FI = high or FI = low indicates FI is in the upper 19% or in the lower 43% of its range. These percentages correspond to breakpoints in the response ranges of each element. In all cases the five input substances (Leptin, Ghrelin, Glucose, CCK, and FHT) are normal but AgRP is inactive while POMC activity is greater than its baseline. The temporal-logic analysis results reported in this table are the same for all model parameterizations: Average, Near, Middle, and Far*.

In temporal logic, the logical implication, if true, means that the consequent occurs *whenever* the antecedent occurs. In the first two rows of Table 3 there are no conditions on NT, OX, or OXT, so these two rows essentially check the proposition that AgRP-inactive and POMC-active, by themselves, determine whether FI is high or low. Those propositions are false, meaning that other units are involved in determining FI, as expected. The rest of Table 3 (rows 3–13) tests propositions involving conditions on the activities of NT, OX, and/or OXT, and the upshot of the analysis, shown in the last row (row 13), is that FI is high with AgRP-inactive and POMC-active as long as NT is active (at any level), OX is active above 20%, and OXT is inactive. These temporal-logic results lead to the model prediction that, when feeding occurs despite low AgRP neuron activity and high POMC neuron activity, NT and OX neurons are active but OXT neurons are inactive (see Discussion).

### Model analysis: focus on GABAergic neurons in LH

The literature describes multiple subtypes of GABAergic neurons in the LH (LH GABAergic neurons), but a consensus on their respective roles in food-intake control has yet to emerge and there is still much disagreement between different studies (Stanley et al., 2011; Laque et al., 2013; Goforth et al., 2014; Jennings et al., 2015; Wu et al., 2015). A great challenge in understanding the contribution of GABAergic neurons in LH is the co-expression (with GABA) of other neuropeptides such as NT, galanin, and MCH, even among LepRB expressing LH GABAergic neurons, combined with the difficulty in distinguishing neuronal subpopulations in LH as compared with other brain regions involved in food-intake regulation. The role of NT-expressing LH GABAergic neurons is especially enigmatic.

The projections of NT-expressing LH GABAergic neurons (NT neurons for short) activate VTA neurons, leading to increased dopamine concentrations in NAc, and this NT neuron projection seems to produce reward since mice will self-administer NT (Kempadoo et al., 2013; Patterson et al., 2015). Release of dopamine into NAc is associated with increased feeding (Hoebel et al., 1992; Volkow et al., 2002) leading to the view that activation of NT neurons should increase food intake. Corroborating this view is the finding that systemic leptin administration, which decreases food intake, also decreases NT expression in LH (Richy et al., 2000). These results indicate that activation of NT-expressing LH GABAergic neurons should increase food intake. However, other findings indicate the opposite.

Administration of an NTR1 agonist decreases food intake (Feifel et al., 2010), while NTR1 knockout increases food intake (Opland et al., 2013). These results suggest that the action of NT (neurotensin), which is released from NT neurons, is to decrease food intake. Other studies show that GABAergic subpopulations of LepRB-expressing LH neurons, which includes those that co-express galanin (i.e., LHGal neurons), but also some that co-express both NT and galanin, inhibit the activity of OX neurons and thereby suppress feeding (Leinninger et al., 2009, 2011). Interestingly, this suppression is GABA independent (Goforth et al., 2014) and is probably mediated by galanin (Laque et al., 2013). In contrast to the results described in the previous paragraph, the results presented in this paragraph indicate that activation of NT-expressing LH GABAergic neurons should decrease food intake.

**Figure 6:**
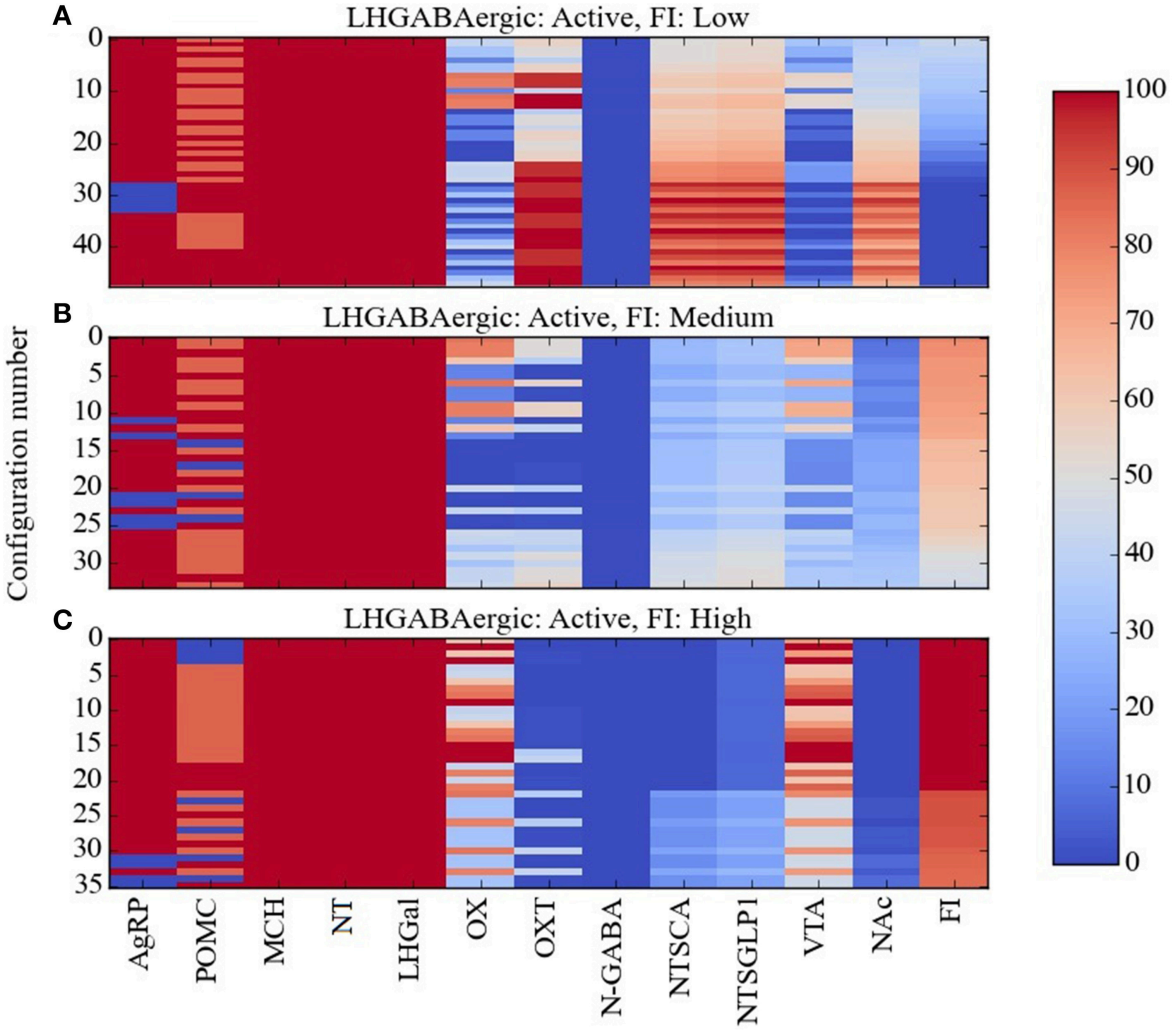
**Percent activity of network units for the same level of activity of LHGABAergic units**. Response configurations corresponding to **(A)** low, **(B)** moderate, and **(C)** high food intake are shown when the LHGABAergic units MCH, NT, and LHGal are all active. Food intake levels at or below 43%, between 44 and 80%, and above 81% are considered low, medium and high, respectively (corresponding to break points in the FI range). Note that the MCH/NT/LHGal/FI pattern in **(A)** is expected but the patterns in **(B,C)** are anomalous (unexpected, paradoxical).

Taken together, the experimental data described previously on the three GABAergic subtypes in LH (the LH GABAergic neurons: MCH, NT, and LHGal) present a perplexing picture. All three should be active together under conditions of satiety, since NT and LHGal are activated by leptin while MCH is activated by glucose, and so they would be expected to suppress food intake. Indeed, LHGal neurons, and the subset of NT neurons that also express galanin, inhibit OX neurons and thereby suppress feeding. Also, the overall action of neurotensin is to suppress feeding. However, activation of MCH neurons promotes feeding by inhibiting OXT and NAc neurons, and some evidence suggests that activation of NT neurons can activate VTA neurons and also promote feeding. In our effort to alleviate some of this confusion, we used Maude to search the model for unit response configurations in which all of the LHGABAergic units in the model were active and in which food intake was low, medium, or high. The three levels chosen corresponded to break points in the distribution of food-intake values produced by the model over the various network configurations and broke down as follows: low, 0–43%; medium, 44–80%; high, 81–100% of maximal food intake achieved by the model. These configurations are shown as heat maps in Figure 6.

Given that the preponderance of the data links activation of LH GABAergic neurons and food-intake suppression, it was not surprising that most of the network response configurations produced by the model were compatible with activation of the three LHGABAergic subtypes (MCH, NT, and LHGal) and decreased food intake. The numbers of network response configurations compatible with LHGABAergic-high/FI-low were 48, 44, 48, and 40 for the Average, Near, Middle and Far parameter cases, respectively (Figure 6A). However, there were also many response configurations compatible with activation of the three LHGABAergic subtypes and medium or high food intake. Specifically, the numbers of configurations compatible with LHGABAergic-high/FI-medium were 34, 48, 27, and 35 (Figure 6B), and with LHGABAergic-high/FI-high were 36, 30, 43, and 43 (Figure 6C). Our interpretation of these modeling results is that there are at least some (perhaps many) response configurations in the real food-intake control system in which high LH GABAergic neuron activity can be associated with low, medium, or high levels of food intake (see Discussion).

**Figure 7:**
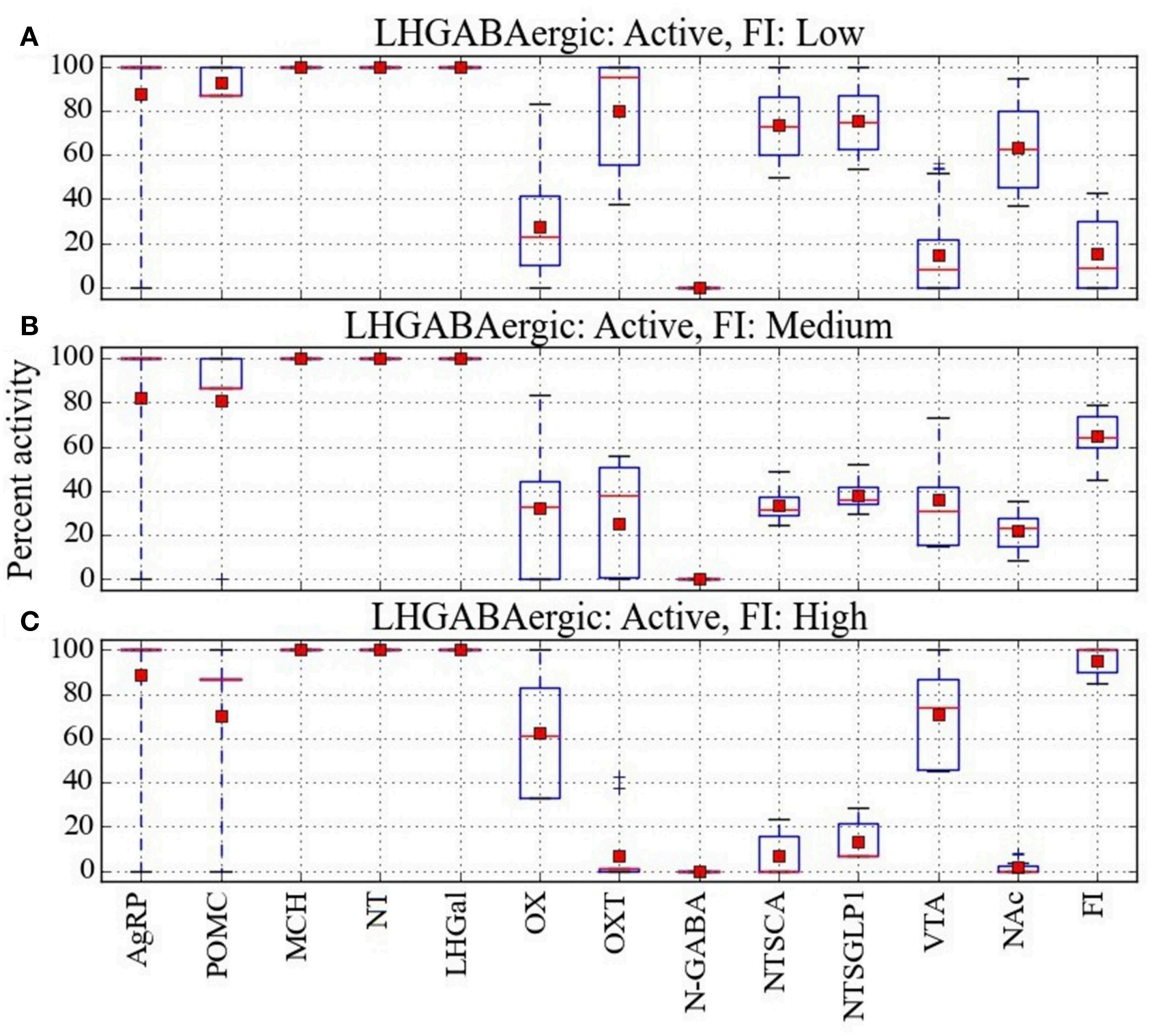
**Analysis of the percent activity for the same level of activity of LHGABAergic units**. Mean activity of each network unit taken over all configurations in cases where activation of all three LHGABAergic units (MCH, NT, and LHGal) is associated with **(A)** low, **(B)** medium, or **(C)** high food intake. Red squares and lines indicate mean and median, respectively. Blue boxes and bars indicate the interquartile range and the entire range of data, respectively. From the mean values it seems that the anomalous patterns, in which activation of MCH, NT, and LHGal is associated with high FI, could occur when OXT is low and OX is high.

In comparing the heat maps in Figures 6A–C, it appears that the three different levels of food intake observed with all LHGABAergic units (MCH, NT, and LHGal) active can be distinguished by different activity levels of units OX and OXT. To facilitate this comparison, the mean activity of all units in the Average parameter case over all configurations are shown for the LHGABAergic-high/FI-low, LHGABAergic-high/FI-medium, and LHGABAergic-high/FI-high patterns in Figures 7A–C, respectively. These show for the Average parameter case that what distinguishes lower from higher levels of food intake are decreased OX and increased OXT activities, corroborating visual inspection of the heat maps (Figures 6A–C). The model suggests that part of the explanation for the finding that activity of LH GABAergic neurons can be associated with high food intake is that it occurs when certain factors limit neural subtype responses so as to increase OX activation but decrease OXT activation (see also Discussion).

Temporal-logic analysis can be used to get a more precise understanding of the conditions that must prevail in order for the model to produce a high level of food intake (FI in the upper 19% of its activity range) even when all three LHGABAergic units (MCH, LHGal, and NT) are fully active. The analysis concerns OX and OXT, which were the units identified from the state-space search results reported above as potentially permissive of the anomalous LHGABAergic-high/FI-high activity pattern. The results of the temporal-logic analysis for this case are presented in Table 4. All of the logical propositions tested are in the form of implications in which the antecedent involves some conditions on the activity of OX and OXT (or no condition, NC), while the consequent is high or low FI. In all cases tested MCH, LHGal, and NT are fully active. The temporal logic results reported in Table 4 are the same for all model parameterizations: Average, Near, Middle, and Far.

**Table 4:** **Temporal-logic analysis: focus on LHGABAergic network units**.

	**Antecedent**	**Consequent**	**Value**
1	OX = NC and OXT = NC	Implies that FI = high	False
2	OX = NC and OXT = 0	Implies that FI = high	False
3	OX = high and OXT = NC	Implies that FI = high	False
4	OX = high and OXT = 0	Implies that FI = high	True
5	OX = NC and OXT = NC	Implies that FI = low	False
6	OX = 0 and OXT = NC	Implies that FI = low	False
7	OX = NC and OXT = high	Implies that FI = low	False
8	OX = 0 and OXT = high	Implies that FI = low	True

*In this table NC stands for no condition, OX, high; OXT, high; FI, high indicates OX, OXT, and FI are in the upper 42, 51, and 19% of their activity ranges, respectively. FI = low indicates that FI is in the lower 43% of its range. These percentages correspond to breakpoints in the response ranges of each element. In all cases the five input substances (Leptin, Ghrelin, Glucose, CCK, and FHT) are normal but the three LHGABAergic neurons (MCH, NT, and LHGal) are active at 100%. The temporal-logic analysis results reported in this table are the same for all model parameterizations: Average, Near, Middle, and Far*.

In the first row of Table 4 there are no conditions on OX or OXT, so this row essentially checks the proposition that MCH, LHGal, and NT fully active, by themselves, determine that FI is high. This proposition is false, meaning that other units are involved in determining FI, as expected. The next three rows (rows 2-4) of Table 4 tests propositions involving conditions on the activities of OX and/or OXT and high FI, which is the anomalous food-intake outcome given MCH, LHGal, and NT fully active. The upshot is that FI is high with MCH, LHGal, and NT fully active (the anomalous outcome) as long as OX is active at a high level (i.e., more than 30% higher than its baseline, which is in the upper 42% of the OX activity range) and OXT is inactive. The second half of Table 4 (rows 5–8) repeats the analysis for FI low, which is the expected food-intake outcome given MCH, LHGal, and NT fully active. The analysis shows that FI is low with MCH, LHGal, and NT fully active (the expected outcome) as long as OX is inactive and OXT is active at a high level (i.e., more than 30% higher than baseline, which is in the upper 51% of the OXT activity range). These temporal-logic results lead to the model prediction that, when feeding occurs despite high activity of the GABAergic neurons in LH, OX neurons are active but OXT neurons are inactive (see Discussion).

## Discussion

Far from being regulated by a simple on-off switch, food-intake control involves a complicated interaction between many different neural subtypes. Our model addresses two specific aspects of the neurobiology of food-intake control. It shows that different and even opposing patterns of AgRP and POMC neuron activity can lead to the same food-intake level and, conversely, the same pattern of activity of GABAergic neurons in LH (MCH, NT, and LHGal all active) can lead to different food-intake levels. While all of the units in the model are involved, our analysis revealed a potentially critical role for NT, OX, and OXT neurons. Our analysis identified OX neurons as permissive of feeding even under circumstances generally associated with food-intake suppression including low AgRP neuron activity combined with high POMC neuron activity, or high activity of LH GABAergic neurons. The model suggests that activation of orexin neurons is essential for the induction of high levels of food intake. This suggestion is consistent with a recent study showing that orexin neuron activity is increased in mice fed a high-fat diet, and such activity is required for their continued high-fat feeding (Valdivia et al., 2014). Conversely, our analysis identified OXT neurons as essential to the ability of POMC neurons to inhibit food intake, and that high levels of food intake do not occur when OXT neuron activity is high. It also suggests that LH GABAergic neurons modulate food intake, at least in part, through their actions on OX and OXT neurons. Some experimental findings are consistent with these modeling results (Sakurai et al., 1998; Brown et al., 2015).

The model is thorough but still incomplete. We use this incompleteness as an advantage in model analysis and interpret the various network response configurations as those that result from limitations in the responses of neural subtypes in the network by neurons outside the network. This approach is highly plausible because it is parsimonious. Obviously, the responses of neurons in the food-intake control network can be modulated by external influences in myriad ways. We restrict the network response configurations we consider to those that represent interruptions in the responses that would otherwise occur as a result of well-documented interactions.

A further restriction we impose is to confine the subset of possible network response configurations we consider to those that result only from changes in the responses of the hypothalamic units. Clearly, changes in the responses of the non-hypothalamic units could alter food intake, the more so because they impinge directly on the food-intake element of the model. By restricting allowed response changes only to hypothalamic units, we ensure that the effect of any change on food intake is not masked simply by failure of a non-hypothalamic unit to update. Of course, external influences could alter food intake by modulating the non-hypothalamic neural subtypes in the network as well, but we avoid those because, in the model, the non-hypothalamic units are the ones that directly impinge on the food-intake element. Network response configurations that include changes in the non-hypothalamic units, specifically those in NTS, NAc, and VTA, would be best considered in models that had other neural types intervening between them and food intake. Those pathways are not included in this initial model because they have yet to be characterized experimentally.

Despite restricting analysis to well-documented interactions and model states involving only hypothalamic neural subtypes, the model reveals a rich set of network response configurations. This computational restriction implies that the real food-intake control network should have many more network response configurations, and they could control food intake under various conditions and according to many different behavioral exigencies. These could include immediate or anticipated energy needs, resource availability, specific nutrient requirements, toxin avoidance, competition for food, predator avoidance, time of day, stress, fear, and even social factors (Woods and Strubbe, 1994; Woods et al., 1998). One challenge going forward is to identify the actual response configurations of the food-intake control network and match them to feeding under different circumstances.

Our model is the only computational model in the food-intake control field that has been specified as a program written in a declarative programming language and analyzed using the techniques of state-space search and temporal-logic model-checking. The model represents the interactions between most of the neural subtypes known to be involved directly in normal food-intake regulation, and model behavior is consistent with many experimental findings. In aggregating current knowledge into an analyzable computational format, the model allows us to explore the implications of that knowledge and to suggest fruitful targets for further research. Because the model represents the most well-established facts on food-intake control proceeding from experiments done so far, its predictions indicate important experiments to do next, given those facts.

As far as we are aware, our model incorporates more of the neural subtypes known to be involved in food-intake control than any other existing model. Still, we realize that this initial model is limited. We excluded some neural subtypes because their connections with other subtypes have yet to be characterized (e.g., VMH, see section Neurobiological Basis of the Model). We also generalized over some potentially relevant factors. For example, multiple receptor subtypes are known to exist for many neurotransmitters and neurohormones, but we represent several of these as generic receptors in the model (e.g., CCKR, DR, GABAR, GalR, OXR, NTR). Similarly, peptides can exist in multiple modified forms (e.g., ghrelin or acyl-ghrelin), but we represent them as generic peptides (e.g., ghrelin). We also generalized over other factors including rodent species/strain, age, sex, and some experimental conditions. These generalizations were necessitated by the variability in preparations that are employed in experiments on food-intake control. Our initial model therefore represents a tradeoff between inclusiveness and specificity, but the modeling approach we have adopted is easily extendable and additional subtypes (of neurons, receptors, etc.) can be added as the model is further developed. As it stands, our initial model provides potential insight into how a set of well-studied hypothalamic neural subtypes might work together to determine the level of food intake. As for any model, whether or not our initial model provides actual insight depends on whether or not its predictions are valid.

The model is based directly on data and it is testable using the same experimental techniques that were used to generate the data on which it is based. Ideally, this research would initiate a computational-experimental interaction in which model predictions are tested, the results are used to correct and expand the model, further predictions are generated and tested, and the cycle continues, producing a model of ever increasing explanatory value. The initiation of such a computational-experimental interaction is the real goal of this paper.

## Author contributions

ST: literature review, computer programming, simulations, analysis, figure and table preparation, and text writing. TA: project conception and design, project direction, programming support, program debugging, and text writing.

### Conflict of interest statement

The authors declare that the research was conducted in the absence of any commercial or financial relationships that could be construed as a potential conflict of interest.
