# Supplementary material for: Computational Analysis of the Hypothalamic Control of Food Intake
Source: Front Comput Neurosci. 2016 Apr 26;10:27. doi: 10.3389/fncom.2016.00027 (PMC4844610; doi:10.3389/fncom.2016.00027)
Supplement: Supplementary file 1 [file Presentation1.PDF]

Supplementary Material

Computational Analysis of the Hypothalamic Control of Food Intake

Shayan Tabe-Bordbar, Thomas J. Anastasio*

Computational Neurobiology Laboratory, Beckman Institute, Department of Molecular and Integrative Physiology, University of Illinois at Urbana-Champaign, Urbana, IL 61801, USA

*** Correspondence:** Thomas J. Anastasio, Beckman Institute, Department of Molecular and Integrative Physiology, University of Illinois at Urbana-Champaign, Urbana, IL 61801, USA

1 Supplementary Data

Numerical data corresponding to GA optimized parameters and parameter correlation analysis, and network response analysis in the Average, Near, Middle, and Far optimized parameter cases, are provided in supplementary data excel files named "Supplementary data S***X***.xlsx" where ***X*** is the number signifying the specific supplementary data mentioned in the following paragraph.

The numerical data for all 43 optimized parameter sets are given in Supplementary Data S1, and pairwise correlation analysis data of the optimized parameters is provided in Supplementary Data S2. The percent activity data for the AgRP-POMC paradox for the Average (corresponding to Figure 4), Far, Middle, and Near cases is given in Supplementary Data S3, S4, S5, and S6, respectively. The percent activity data for the LH GABAergic conundrum for the Average (corresponding to figure 6), Far, Middle, and Near cases are provided in supplementary data S7, S8, S9, and S10, respectively. Percent activity of all unique response configurations for the Average, Far, Middle, and Near cases is given in supplementary data S11.

2 Supplementary Figures and Tables

In the main article, Figures 4 through 7 show percentage responses in the Average case. Figures corresponding to Figures 4 through 7 for Near, Middle, and Far parameter cases are provided in the following supplementary figures. The complete list of all model parameters is provided in the Supplementary table.

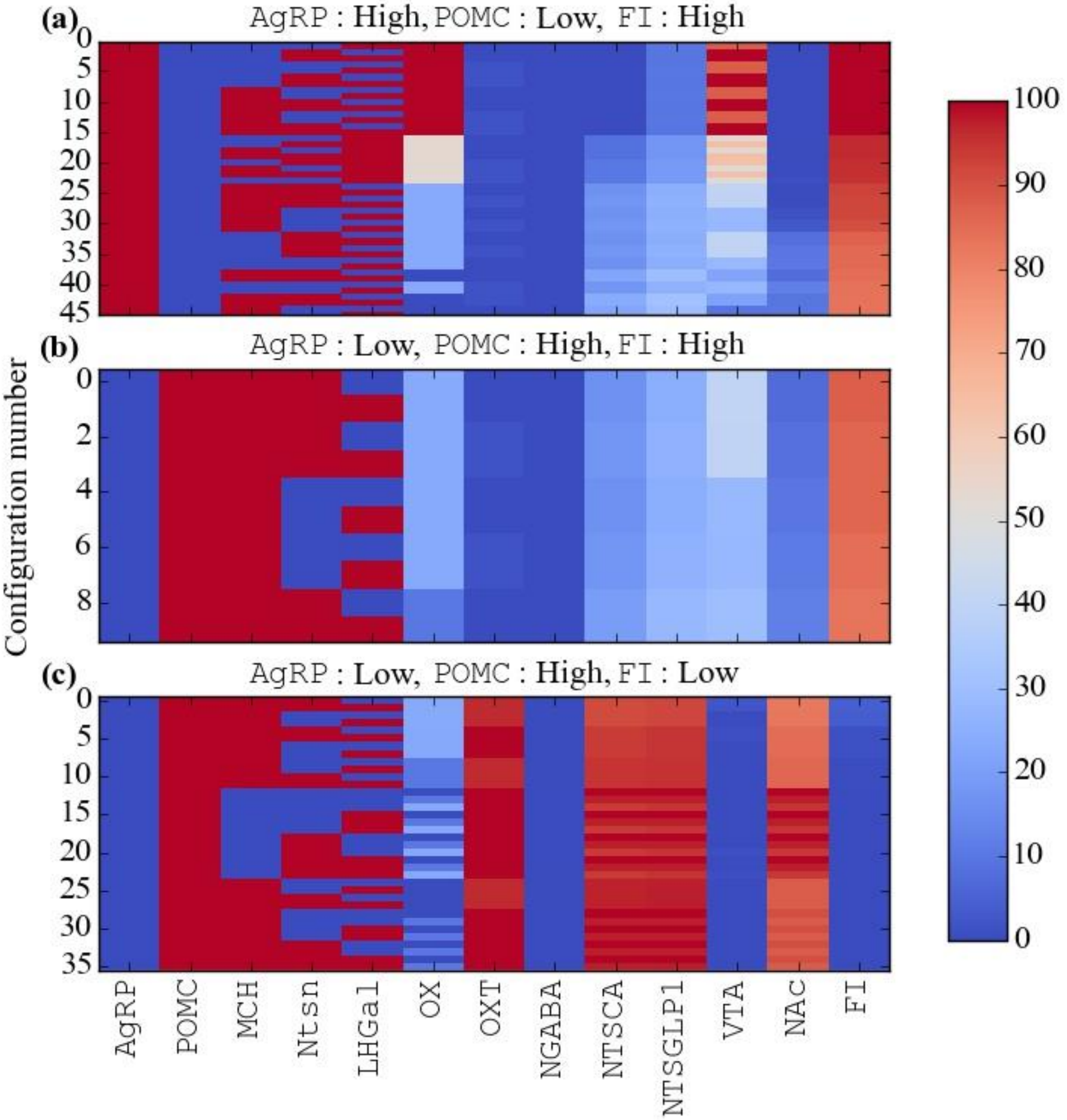

Supplementary Figure S1. Percent activity of network units for different patterns of `AgRP` and `POMC` activity in the Far case. Response configurations corresponding to high food intake are shown when (**a**) `AgRP` is active and `POMC` is inactive, or (**b**) when `AgRP` is inactive and `POMC` is active. Response configurations corresponding to low food intake are shown when (**c**) `AgRP` is inactive and `POMC` is active. Food-intake levels above 81% are considered high while those below 43% are considered low (corresponding to break points in the `FI` range). Note that the `AgRP/POMC/FI` patterns in (**a**) and (**c**) are expected but the pattern in (**b**) is anomalous (unexpected, paradoxical).

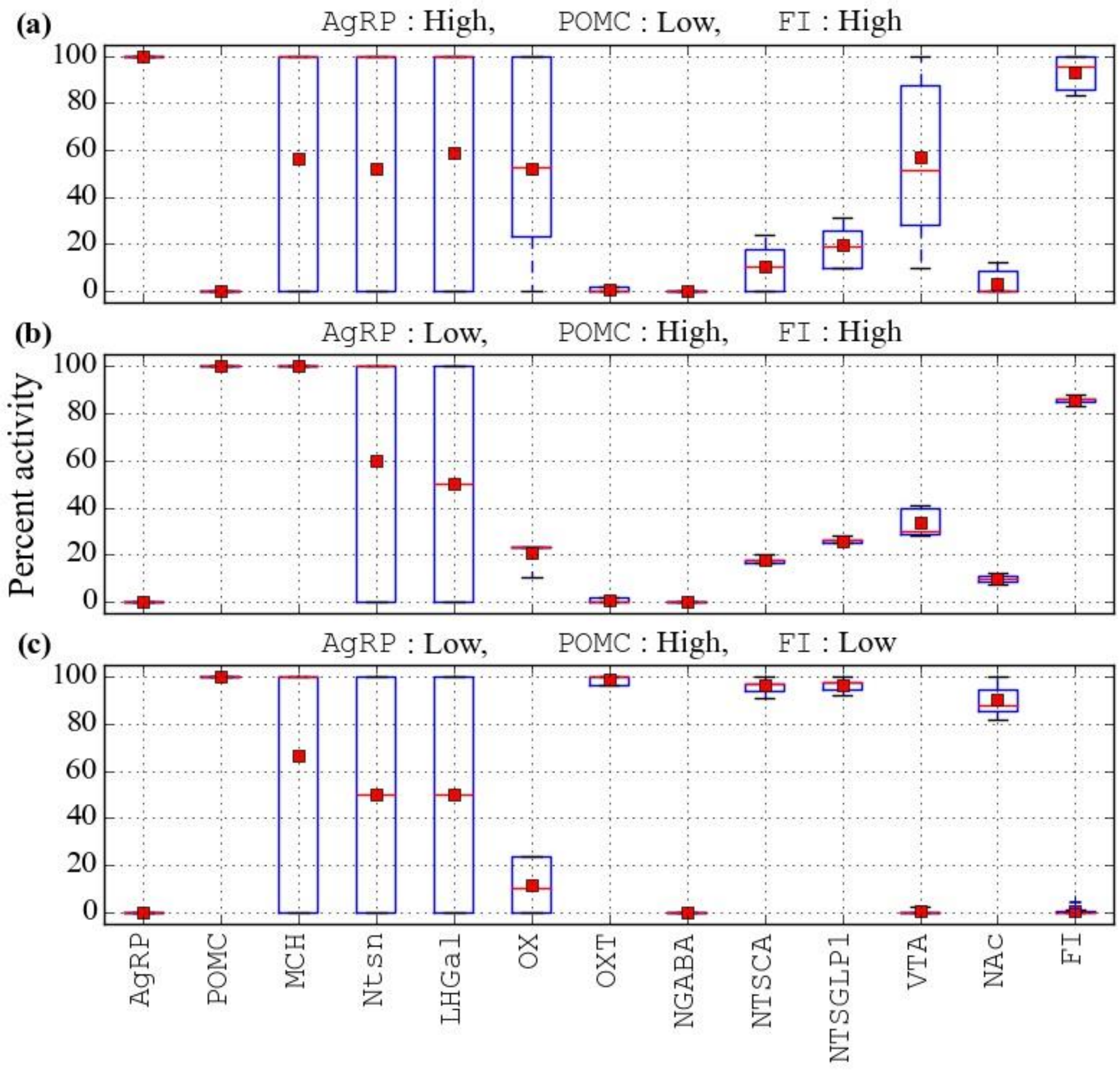

Supplementary Figure S2. Analysis of the percent activity for different patterns of `AgRP` and `POMC` activity in the Far case. Mean activity of each network unit taken over all configurations in cases where (**a**) activation of `AgRP` and inactivation of `POMC` is associated with high food intake, (**b**) inactivation of `AgRP` and activation of `POMC` is associated with high food intake, and (**c**) inactivation of `AgRP` and activation of `POMC` is associated with low food intake. Red squares and lines indicate mean and median, respectively. Blue boxes and bars indicate the interquartile range and the entire range of data, respectively.

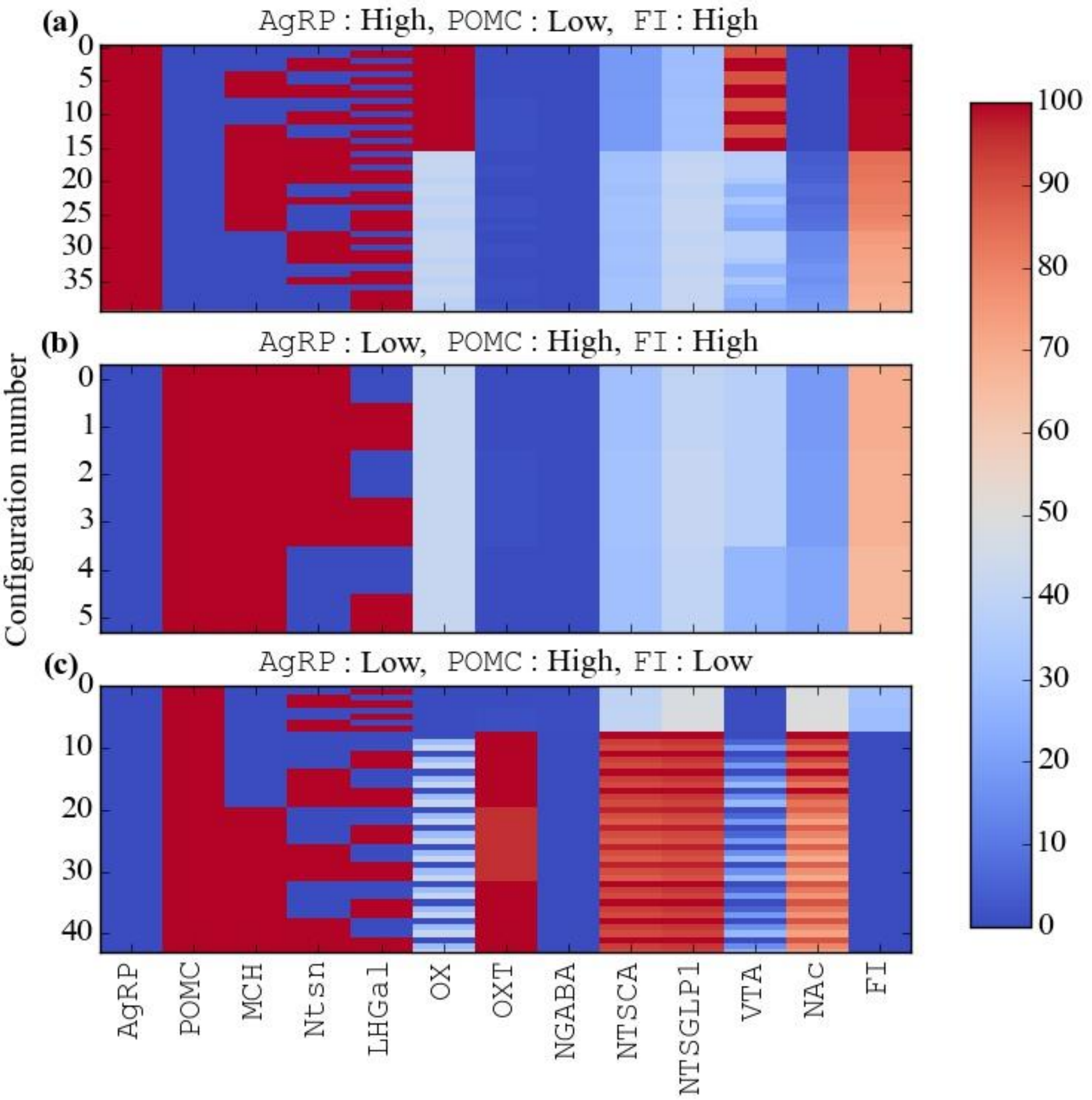

Supplementary Figure S3. Percent activity of network units for different patterns of `AgRP` and `POMC` activity for the Middle case. Response configurations corresponding to high food intake are shown when (**a**) `AgRP` is active and `POMC` is inactive, or (**b**) when `AgRP` is inactive and `POMC` is active. Response configurations corresponding to low food intake are shown when (**c**) `AgRP` is inactive and `POMC` is active. Food-intake levels above 81% are considered high while those below 43% are considered low (corresponding to break points in the `FI` range). Note that the `AgRP`/`POMC`/`FI` patterns in (**a**) and (**c**) are expected but the pattern in (**b**) is anomalous (unexpected, paradoxical).

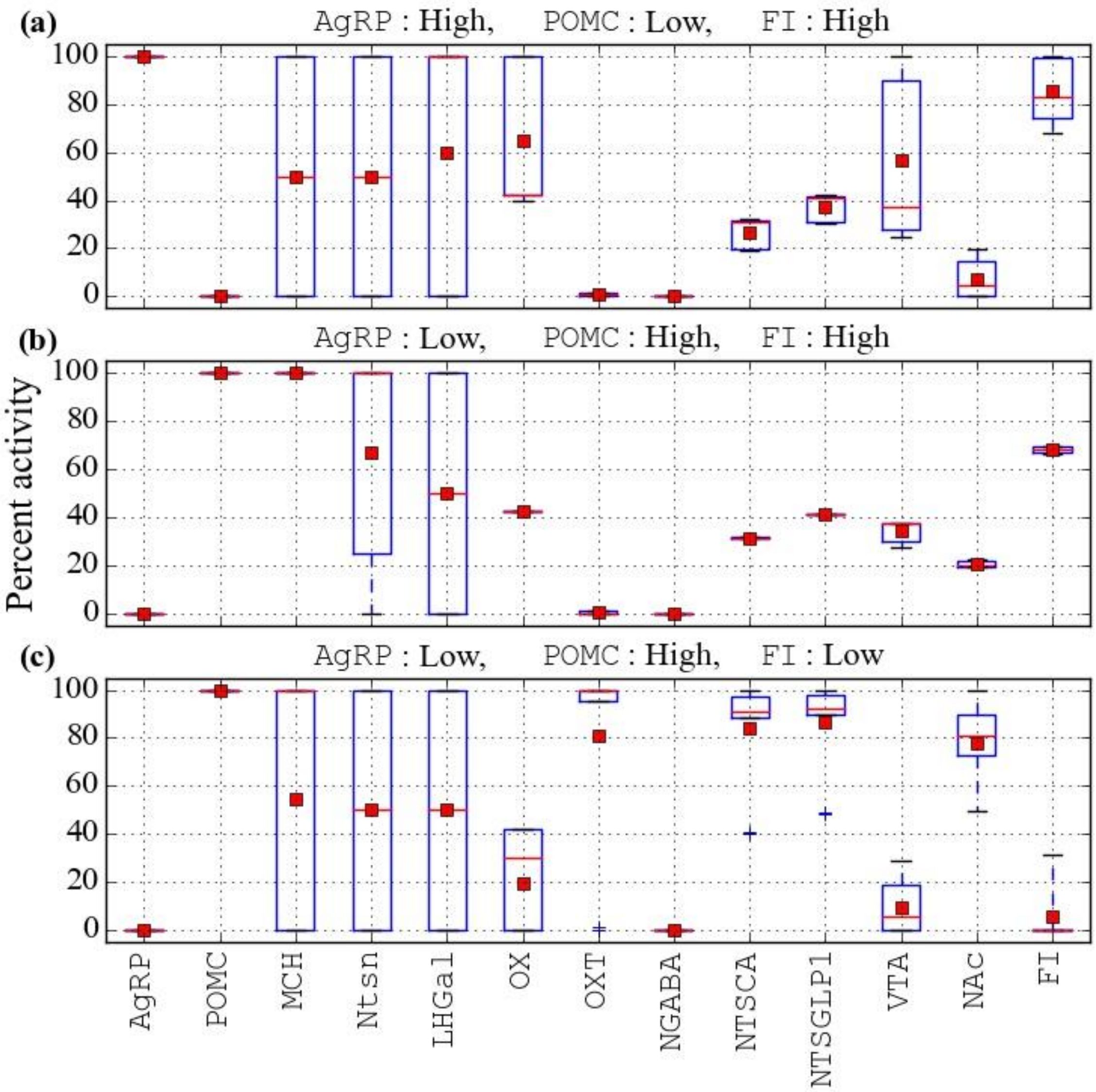

Supplementary Figure S4. Analysis of the percent activity for different patterns of AgRP and POMC activity in the Middle case. Mean activity of each network unit taken over all configurations in cases where (**a**) activation of AgRP and inactivation of POMC is associated with high food intake, (**b**) inactivation of AgRP and activation of POMC is associated with high food intake, and (**c**) inactivation of AgRP and activation of POMC is associated with low food intake. Red squares and lines indicate mean and median, respectively. Blue boxes and bars indicate the interquartile range and the entire range of data, respectively.

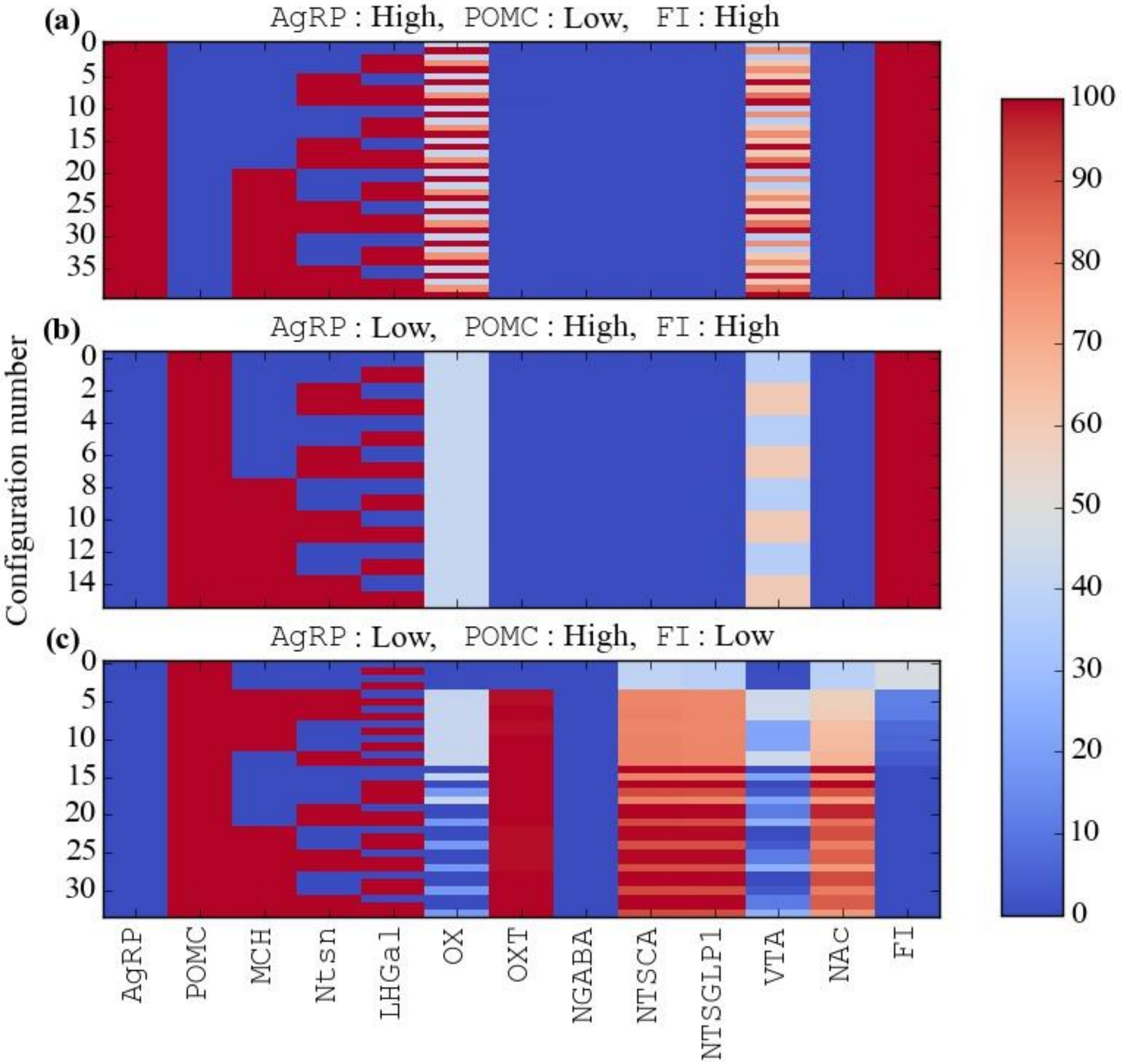

Supplementary Figure S5. Percent activity of network units for different patterns of `AgRP` and `POMC` activity for the Near case. Response configurations corresponding to high food intake are shown when (**a**) AgRP is active and POMC is inactive, or (**b**) when AgRP is inactive and POMC is active. Response configurations corresponding to low food intake are shown when (**c**) AgRP is inactive and POMC is active. Food-intake levels above 81% are considered high while those below 43% are considered low (corresponding to break points in the FI range). Note that the AgRP/POMC/FI patterns in (**a**) and (**c**) are expected but the pattern in (**b**) is anomalous (unexpected, paradoxical).

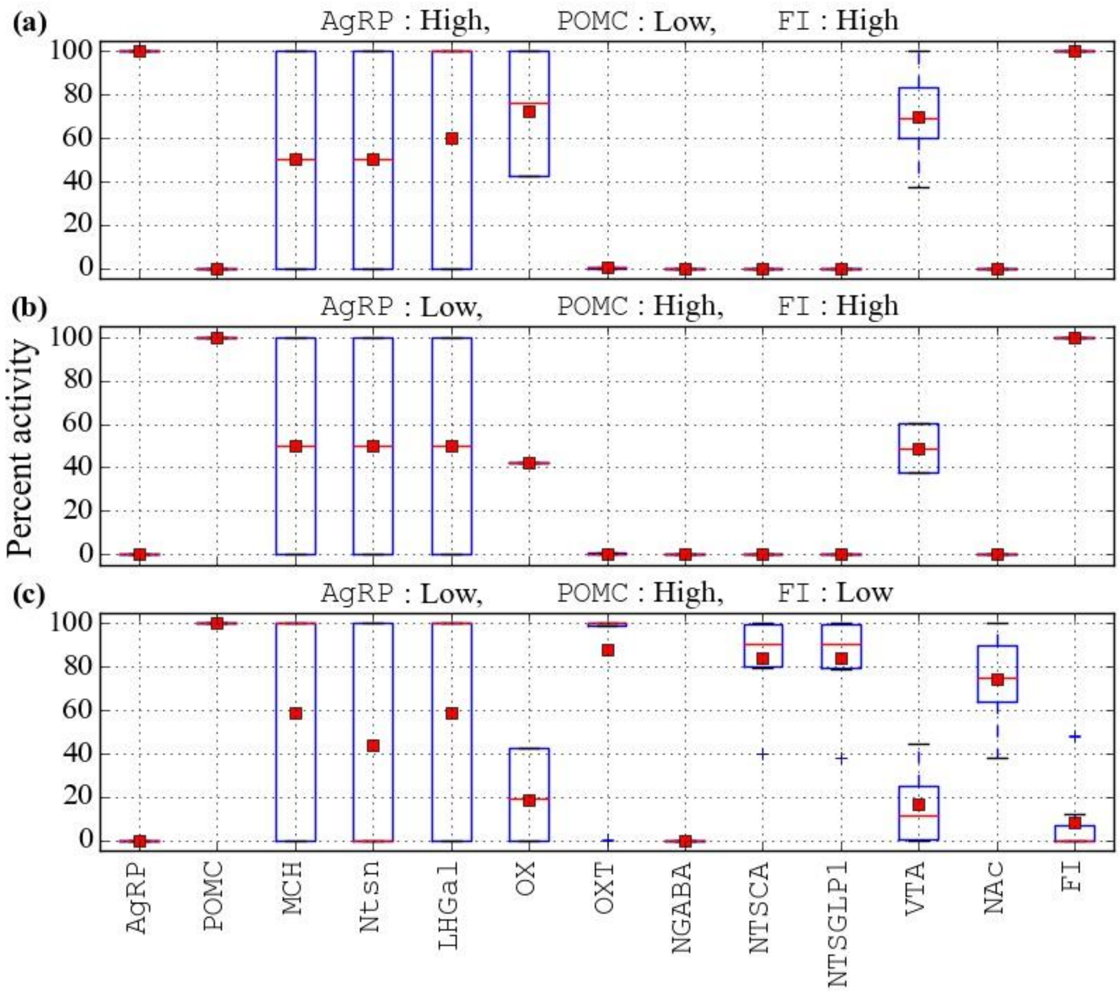

Supplementary Figure S6. . Analysis of the percent activity for different patterns of AgRP and POMC activity in the Near case. Mean activity of each network unit taken over all configurations in cases where (**a**) activation of AgRP and inactivation of POMC is associated with high food intake, (**b**) inactivation of AgRP and activation of POMC is associated with high food intake, and (**c**) inactivation of AgRP and activation of POMC is associated with low food intake. Red squares and lines indicate mean and median, respectively. Blue boxes and bars indicate the interquartile range and the entire range of data, respectively.

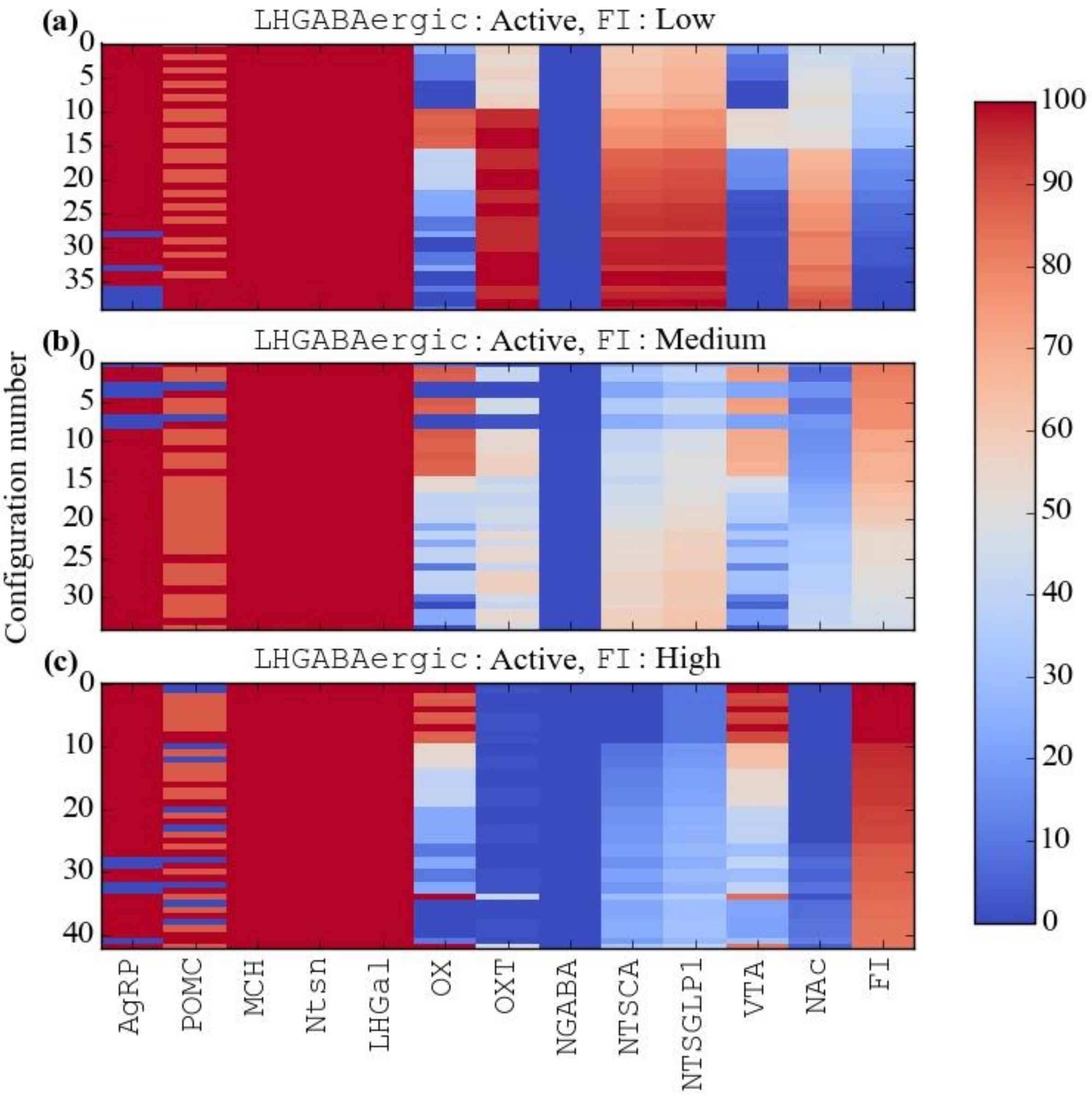

Supplementary Figure S7. Percent activity of network units for the same level of activity of `LHGABAergic` units in the Far case. Response configurations corresponding to (**a**) low, (**b**) moderate, and (**c**) high food intake while the `LHGABAergic` units `MCH`, `Ntsn`, and `LHGal` are all active. Food intake levels between 0 and 43%, between 43 and 81%, and between 81-100% are considered low, medium and high, respectively (corresponding to break points in the `FI` range). Note that the `MCH`/`Ntsn`/`LHGal`/`FI` pattern in (**a**) is expected but the patterns in (**b**) and (**c**) are anomalous (unexpected, paradoxical).

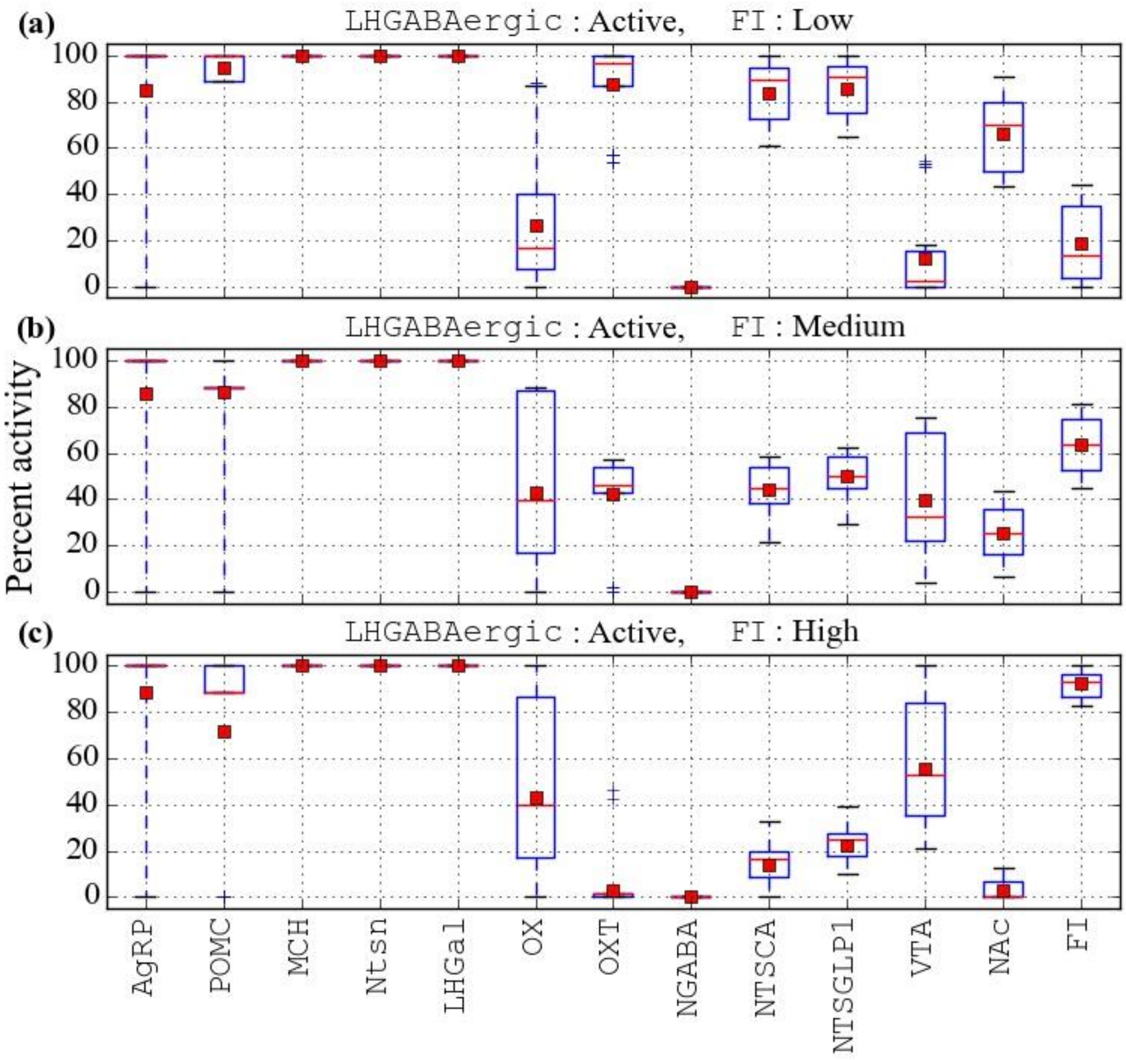

Supplementary Figure S8. Analysis of the percent activity for the same level of activity of `LHGABAergic` units in the Far case. Mean activity of each network unit taken over all configurations in cases where activation of all three `LHGABAergic` units (`MCH`, `Ntsn`, and `LHGal`) is associated with (**a**) low, (**b**) medium, or (**c**) high food intake. Red squares and lines indicate mean and median, respectively. Blue boxes and bars indicate the interquartile range and the entire range of data, respectively.

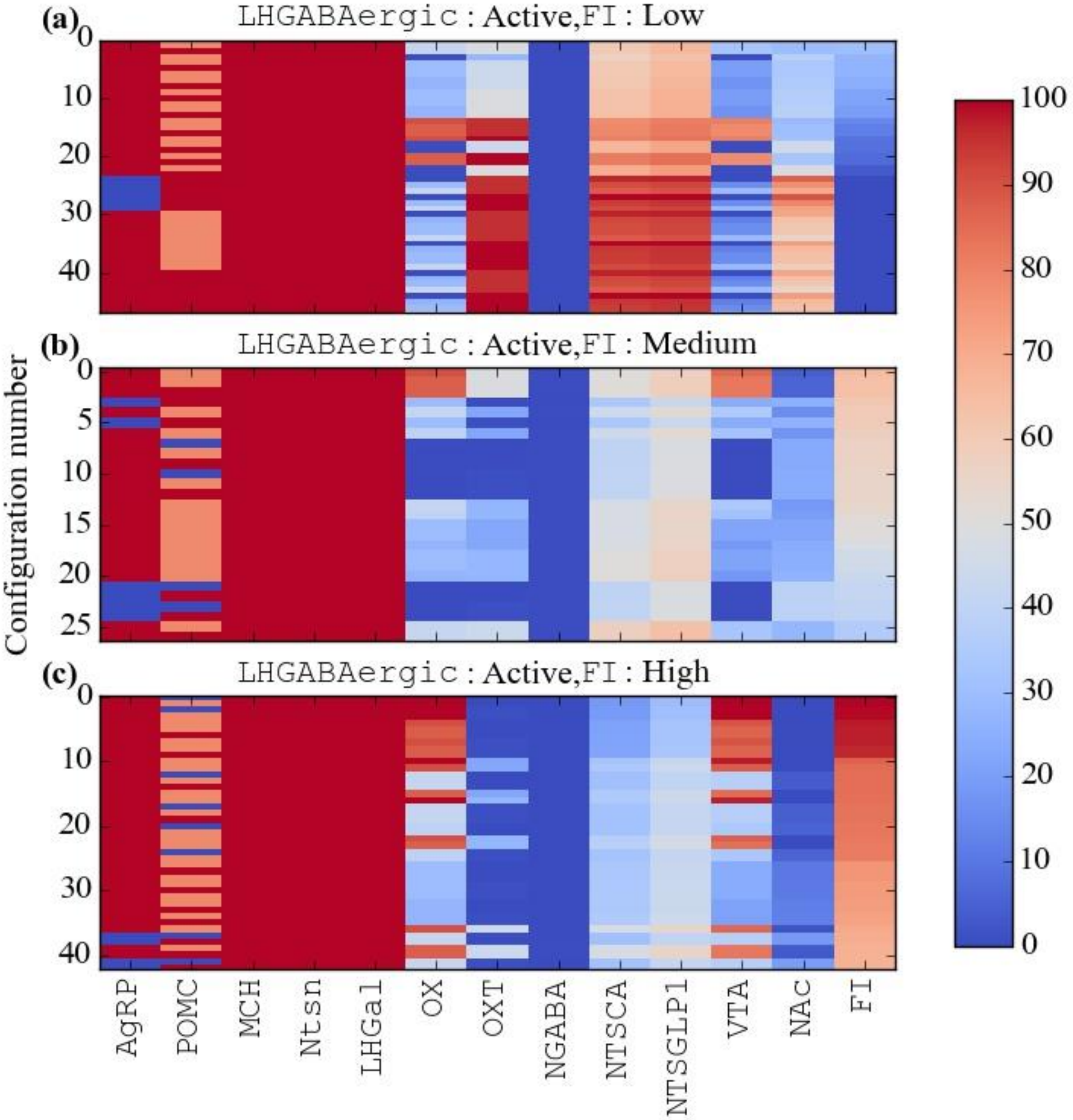

Supplementary Figure S9. Percent activity of network units for the same level of activity of `LHGABAergic` units in the Middle case. Response configurations corresponding to (**a**) low, (**b**) moderate, and (**c**) high food intake while the `LHGABAergic` units `MCH`, `Ntsn`, and `LHGal` are all active. Food intake levels between 0 and 43%, between 43 and 81%, and between 81-100% are considered low, medium and high, respectively (corresponding to break points in the `FI` range). Note that the `MCH`/`Ntsn`/`LHGal`/`FI` pattern in (**a**) is expected but the patterns in (**b**) and (**c**) are anomalous (unexpected, paradoxical).

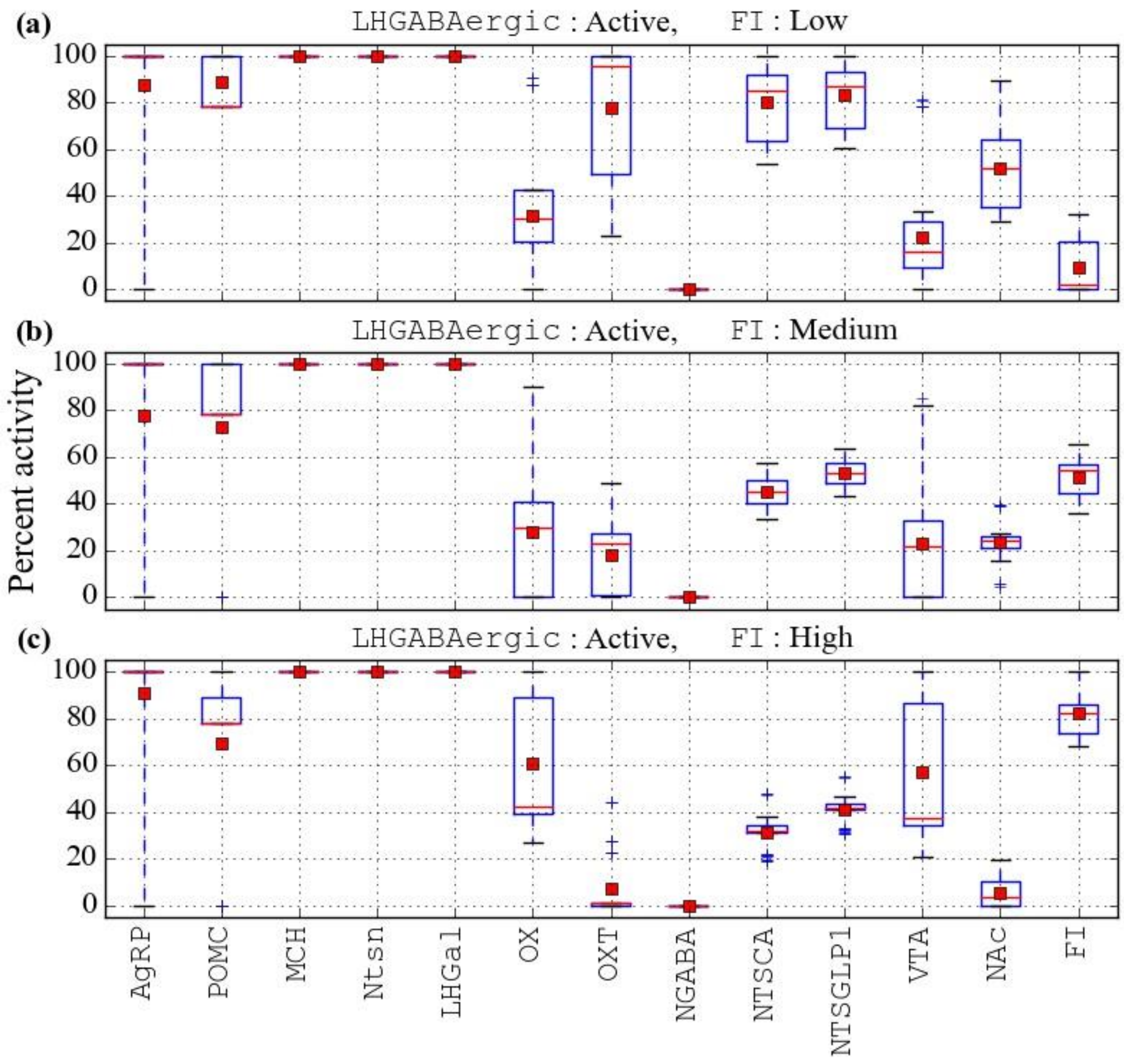

Supplementary Figure S10. Analysis of the percent activity for the same level of activity of `LHGABAergic` units in the Middle case. Mean activity of each network unit taken over all configurations in cases where activation of all three `LHGABAergic` units (`MCH`, `Ntsn`, and `LHGal`) is associated with (**a**) low, (**b**) medium, or (**c**) high food intake. Red squares and lines indicate mean and median, respectively. Blue boxes and bars indicate the interquartile range and the entire range of data, respectively.

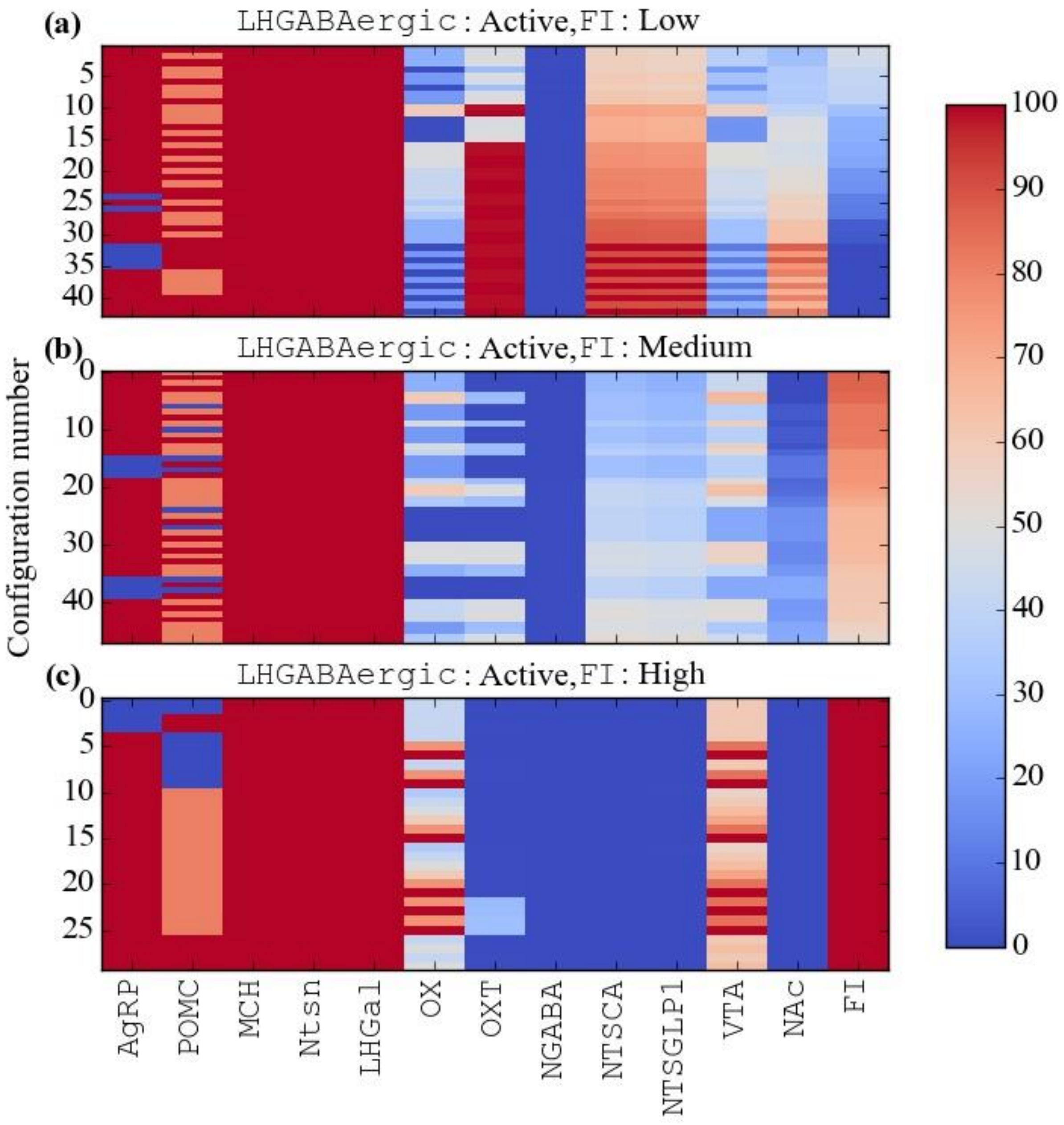

Supplementary Figure S11. Percent activity of network units for the same level of activity of `LHGABAergic` units in the Near case. Response configurations corresponding to (**a**) low, (**b**) moderate, and (**c**) high food intake while the `LHGABAergic` units `MCH`, `Ntsn`, and `LHGal` are all active. Food intake levels between 0 and 43%, between 43 and 81%, and between 81-100% are considered low, medium and high, respectively (corresponding to break points in the `FI` range). Note that the `MCH`/`Ntsn`/`LHGal`/`FI` pattern in (**a**) is expected but the patterns in (**b**) and (**c**) are anomalous (unexpected, paradoxical).

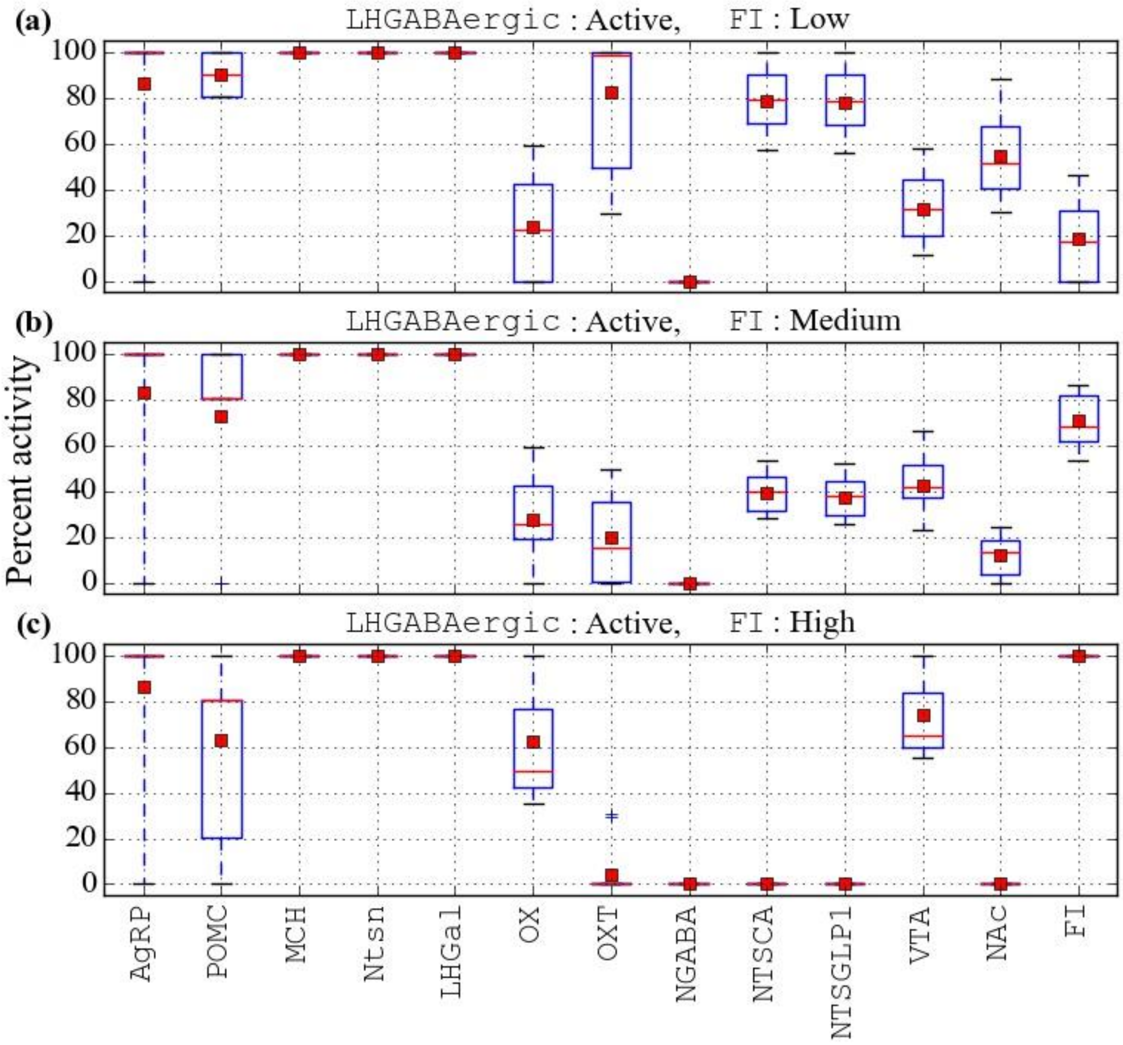

Supplementary Figure S12. Analysis of the percent activity for the same level of activity of `LHGABAergic` units in the Near case. Mean activity of each network unit taken over all configurations in cases where activation of all three `LHGABAergic` units (`MCH`, `Ntsn`, and `LHGal`) is associated with (**a**) low, (**b**) medium, or (**c**) high food intake. Red squares and lines indicate mean and median, respectively. Blue boxes and bars indicate the interquartile range and the entire range of data, respectively.

Supplementary Table S1. Description of model parameters.

Number	Parameter	Unit
0	GHSR	AgRP
1	LepRB	AgRP
2	FHT1BR	AgRP
3	LepRB	N-GABA
4	GABAR	POMC
5	Y1R	POMC
6	FHT2CR	POMC
7	LepRB	POMC
8	GLUR	MCH
9	LepRB	Ntsn
10	LepRB	LHGal
11	MC4R	OXT
12	MC3R	OXT
13	GABAR	OXT
14	Y1R	OXT
15	MCHR	OXT
16	GHSR	OX
17	Y1R	OX
18	GABAR	OX
19	GalR	OX
20	GLUR	OX
21	MC4R	OX
22	OXR	NTSCA
23	OXTR	NTSCA
24	LepRB	NTSCA
25	CCKR	NTSCA

Number	Parameter	Unit
26	GHSR	NTSCA
27	LepRB	NTSGLP1
28	AR	NTSGLP1
29	GHSR	NTSGLP1
30	LepRB	VTA
31	GHSR	VTA
32	OXR	VTA
33	NtsnR	VTA
34	GLP1R	VTA
35	DR	NAc
36	MCHR	NAc
37	GLP1R	NAc
38	Y1R	NAc
39	Bias	AgRP
40	Bias	N-GABA
41	Bias	POMC
42	Bias	MCH
43	Bias	OXT
44	Bias	Ntsn
45	Bias	LHGal
46	Bias	OX
47	Bias	NTSCA
48	Bias	NTSGLP1
49	Bias	VTA
50	Bias	NAc
51	Bias	FI
